# Toward Transition‐Metal‐Templated Construction of Arylated B_4_ Chains by Dihydroborane Dehydrocoupling

**DOI:** 10.1002/chem.201904772

**Published:** 2019-12-09

**Authors:** Carsten Lenczyk, Dipak Kumar Roy, Kai Oberdorf, Jörn Nitsch, Rian D. Dewhurst, Krzysztof Radacki, Jean‐François Halet, Todd B. Marder, Matthias Bickelhaupt, Holger Braunschweig

**Affiliations:** ^1^ Institute for Inorganic Chemistry Julius-Maximilians-Universität Würzburg, Am Hubland, 97074 Würzburg (Germany), and Institute for, Sustainable Chemistry & Catalysis with Boron, Julius-Maximilians-Universität Würzburg Am Hubland 97074 Würzburg Germany; ^2^ Discipline of Chemistry Indian Institute of Technology Indore Khandwa Road, Simrol Indore 453552, M.P. India; ^3^ Univ Rennes CNRS Institut des Sciences Chimiques de Rennes UMR 6226 35000 Rennes France; ^4^ Department of Theoretical Chemistry and Amsterdam Center for, Multiscale Modeling (ACMM) Vrije Universiteit Amsterdam De Boelelaan 1083, 1081 HV Amsterdam (The Netherlands), and Institute for Molecules and Materials (IMM), Radboud University, Heyendaalseweg 135 6525 AJ Nijmegen The Netherlands

**Keywords:** B−H activation, boron, dehydrocoupling, ruthenium, transition metal

## Abstract

The reactivity of a diruthenium tetrahydride complex towards three selected dihydroboranes was investigated. The use of [DurBH_2_] (Dur=2,3,5,6‐Me_4_C_6_H) and [(Me_3_Si)_2_NBH_2_] led to the formation of bridging borylene complexes of the form [(Cp*RuH)_2_BR] (Cp*=C_5_Me_5_; **1 a**: R=Dur; **1 b**: R=N(SiMe_3_)_2_) through oxidative addition of the B−H bonds with concomitant hydrogen liberation. Employing the more electron‐deficient dihydroborane [3,5‐(CF_3_)_2_‐C_6_H_3_BH_2_] led to the formation of an anionic complex bearing a tetraarylated chain of four boron atoms, namely Li(THF)_4_[(Cp*Ru)_2_B_4_H_5_(3,5‐(CF_3_)_2_C_6_H_3_)_4_] (**4**), through an unusual, incomplete threefold dehydrocoupling process. A comparative theoretical investigation of the bonding in a simplified model of **4** and the analogous complex *nido*‐[1,2(Cp*Ru)_2_(μ‐H)B_4_H_9_] (**I**) indicates that there appear to be no classical σ‐bonds between the boron atoms in complex **I**, whereas in the case of **4** the B_4_ chain better resembles a network of three B−B σ bonds, the central bond being significantly weaker than the other two.

## Graphical abstract

**Daisy chains of boron**: Reactions of dihydroboranes with [(Cp*Ru)_2_(μ‐H)_4_] led to complexes arising from differing extents of hydrogen loss, including mono‐ and dinuclear trihydroborate complexes, bridging borylene complexes, and a dinuclear complex bearing a tetraarylated B_4_ unit.

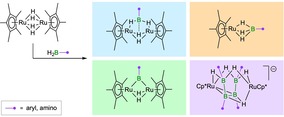

Diborane(4) compounds are highly useful synthetic modules for organic synthesis,^1^ in particular in catalytic diboration^2^ and other borylation reactions.^1^, ^3^ As such, diborane(4) compounds represent the simplest—and by far the best‐known—examples of boron‐chain molecules. Despite their ubiquitous use throughout organic chemistry, only a handful of diboron compounds are commercially available.^1^, ^4^ Longer chains of boron molecules are practically non‐existent. This phenomenon was elegantly summarized by Boldyrev in a 2012 computational study of B_*n*_H_*n*+2_ molecules, which showed that, in the absence of electronic or steric perturbations, the stability of linear chains—that is, those with sp^2^‐hybridized boron atoms and electron‐precise B−B bonds—quickly diminishes relative to cluster structures as *n* increases.^5^ For some time, our groups have been involved in the search for techniques to form electron‐precise B−B bonds selectively, with the ultimate goal of preparing long chains of hypovalent boron atoms. This challenge has resulted in the development of new metal‐free and metal‐mediated B−B bond formation processes, although the synthesis of boron chains remains difficult.^4^, ^6^, ^7^

One promising strategy for the preparation of linear boron chains is their construction on a metal template, followed by demetallation. The construction of linear boron chains as part of multinuclear transition metal clusters, although rare in comparison with more complex clusters of boron atoms, is known in the literature. Typically, these reactions involve the combination and thermolysis of a metal‐halide source, a borohydride salt and/or a BH_3_ adduct, with some reactions also involving a second organometallic fragment.^8^, ^9^, ^10^ This technique, pioneered by the group of Fehlner and since built upon significantly by Ghosh and co‐workers, generally provides products in which the boron atoms are bound exclusively to hydride and metal groups. Examples of clusters containing functionalized B_4_ chains exist in the literature, namely [(Cp*Mo)_2_B_2_H_5_(BER)_2_(μ‐η^1^‐ER)] (E=S, *R*=2,6‐(*t*Bu)_2_‐C_6_H_2_OH; E=Se, R=Ph; Cp*=η^5^‐Me_5_C_5_) and *nido*‐[(Cp*Ru)_2_B_4_H_9_(SePh)], reported by Ghosh and co‐workers, resulting from the application of dichalcogenides or the phenylselenolate‐functionalized salt Li[H_3_B(SePh)] in place of a borohydride salt.^11^ However, despite the presence of a boron‐bound SePh group in the precursor, only one of the boron atoms in the final product was ultimately found to bear this group in the latter case.

Almost all longer linear chains of boron atoms are metal‐free and include π‐donor groups (such as dialkylamino) bound to boron; indeed their presence is likely useful for their formation.^12^ However, the reliance on such groups is expected to reduce the π‐acceptor character of the boron atoms drastically and thus dampen their potentially interesting reactivity and electronic properties. In our attempts to find synthetic strategies to construct chains of boron atoms, we have thus mainly focused on the use of non‐π‐donor substituents at boron.^13^

This work describes our efforts to apply the known metal‐mediated hydroborane dehydrocoupling process^6^ to the construction of chains of hypovalent boron atoms bearing substituents other than hydrogen. We establish herein the exceptional ability of the diruthenium tetrahydrido complex [(Cp*Ru)_2_(μ‐H)_4_] to mediate the dehydrogenation of functionalized dihydroboranes, leading either to bridging borylene complexes or the synthesis of complexes bearing B−B‐bonded ligands. The latter includes the synthesis of a complex bearing a unit containing four connected boron atoms, representing the first example of a perarylated boron chain.

The high electron density of metal hydride clusters makes them potentially very active towards oxidative addition of substrates.^14^ We thus reasoned that the polyhydride complex [(Cp*Ru)_2_(μ‐H)_4_] may favor the activation of functionalized dihydroboranes to produce borylene complexes, in contrast to the mononuclear polyhydride ruthenium complexes [(R_3_P)_2_RuH_2_(H_2_)_2_], which, in our hands, have proven reluctant to dehydrogenate dihydroboranes fully, instead providing bis(σ)‐boranes.^15^

Addition of slight excesses of dihydroboranes (DurBH_2_ and (Me_3_Si)_2_NBH_2_) to THF solutions of [(Cp*Ru)_2_(μ‐H)_4_], and heating of the reaction mixtures at 60 °C, led to color changes of the solutions to yellow, and subsequent isolation of the bridging borylene complexes [(Cp*Ru)_2_(μ‐H)_2_(μ‐BR)] (**1 a**: R=Dur, 11 % yield; **1 b**: R=N(SiMe_3_)_2_, 10 % yield, Scheme 1). These complexes were identified by NMR spectroscopy, mass spectrometry, and single‐crystal X‐ray diffraction analyses. Compounds **1 a** and **1 b** show single broad ^11^B NMR signals at *δ*=127.4 and 91.5 ppm, respectively, both far downfield from that of the (DurBH_2_)_2_ dimer in benzene (*δ*=22.4 ppm) and (Me_3_Si)_2_NBH_2_ (*δ*=46.3 ppm), and consistent with the signals of other bridging borylene complexes.16a, 16b Broad singlets were observed (**1 a**: −12.28; **1 b**: −13.17 ppm) for the bridging Ru−H−Ru hydrides (integral approx. 2 H relative to the Cp* ligand) in the ^1^H NMR spectra of **1 a** and **1 b**. Crystallization at room temperature allowed single‐crystal X‐ray diffraction studies of the complexes, confirming the presence of a borylene (BR) unit bridging the [(Cp*Ru)_2_(μ‐H)_2_] fragments (Figure 1). Interestingly, the short Ru−Ru distance (2.463(1) Å) observed in [(Cp*Ru)_2_(μ‐H)_4_] is further shortened in the borylene complexes **1 a** (2.4220(5)) and **1 b** (2.434(1) Å). The Ru−B bond lengths (**1 a**: 2.049(4), 2.047(4); **1 b**: 2.110(7), 2.095(7) Å) are in the range typical of known transition‐metal‐bridging borylene complexes of di‐ and trinuclear carbonyl‐bridged16c or trinuclear hydride‐bridged16d complexes.

**Scheme 1:**
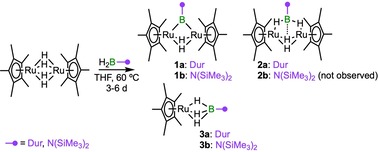
Double B−H activation of a dihydroborane by a dinuclear ruthenium complex, leading to bridging borylene complexes **1 a**,**b**.

**Figure 1:**
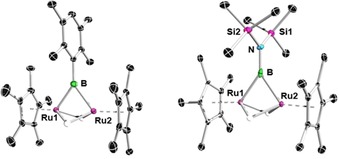
Crystallographically derived structures of bridging borylene complexes **1 a** (left) and **1 b** (right). Ellipsoids shown at the 50 % probability level. All hydrogen atoms bound to carbon atoms have been omitted for clarity. Selected bond lengths (Å) and angles (°) for **1 a**: Ru1−B 2.049(4), Ru2−B 2.047(4), Ru1−Ru2 2.4220(5), B−C1 1.538(5); Ru1‐B‐Ru2 53.8(1). For **1 b**: Ru1−B 2.110(7), Ru2−B 2.095(7), Ru1−Ru2 2.434(1), B−N 1.421(8); Ru1‐B‐Ru2 54.3(2).

Given the isolation of borylene complexes **1 a** and **1 b**, we sought to determine the intermediate(s) formed during the course of the reaction. Recording ^11^B NMR spectra directly after the addition of duryldihydroborane to [(Cp*Ru)_2_(μ‐H)_4_], which is accompanied by immediate gas evolution, indicated the formation of two new compounds (^11^B NMR signals at *δ*=62 and 25 ppm) which were later crystallographically identified as hydroborate complexes [(Cp*Ru)_2_(μ‐H)(μ*‐*κ^3^‐H,H,H‐H_3_BDur)] (**2 a**) and [Cp*Ru{κ^3^‐H,H,H‐(H_3_BDur)}] (**3 a**). Further heating and evaporation of the initial mixture under vacuum resulted in the formation of the bridging borylene complex **1 a**. A small sample of red crystals of **2 a** were isolated, and their solid‐state structure was determined to consist of a DurBH_3_ ligand bridging two Ru centers, with an additional hydride bridging the two Ru centers (Figure 2), structurally analogous to the reported dirhodium complex [{(DiPPE)Rh}_2_(μ‐H){μ‐η^2^‐H_2_BH(CMe_2_ *i*Pr)}] (DiPPE=1,2‐bis(diisopropylphosphino)ethane).^17^ The Ru−Ru distance (2.7307(13) Å) in **2 a** is significantly longer than that in **1 a** (2.4220(5) Å) and [(Cp*Ru)_2_(μ‐H)_4_] (2.463(1) Å). The distance in the former corresponds to that generally observed in complexes with single bonds between ruthenium centers. The Ru−B distances of **2 a** are slightly longer than those of **1 a**.

**Figure 2:**
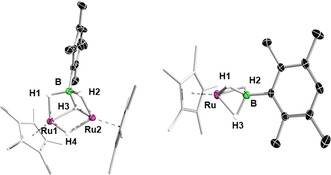
Crystallographically derived structures of bridging trihydroborate complexes **2 a** (left) and **3 a** (right). Ellipsoids shown at the 50 % probability level. All hydrogen atoms bound to carbon atoms have been omitted for clarity. Selected bond lengths (Å) for **2 a**: Ru1−B 2.254(4), Ru2−B 2.184(4), Ru1−Ru2 2.7307(13), Ru1−H1 1.72(4), Ru2−H2 1.66(4), B−H1 1.26(4), B−H2 1.27(4), B−H3 1.45(4). For **3 a**: Ru−B 1.948(4), Ru−H1 1.71(4), Ru−H2 1.79(4), Ru−H3 1.81(4), B−H1 1.29(3), B−H2 1.26(4), B−H3 1.30(4).

Interestingly, in addition to **2 a**, yellow crystals of **3 a** (see Scheme S22, Supporting Information) were also isolated from the slow evaporation of the pentane extract of the above‐mentioned reaction. The solid‐state structure of **3 a** (Figure 2) similarly exhibits a DurBH_3_ ligand, which in this case is bound through all three hydrogens to a single Ru center in a κ^3^ fashion. The Ru−hydrogen distances are similar to those of reported κ^3^‐bound σ‐borate complexes of ruthenium.^18^ Although we were able to isolate **2 a** and **3 a** in the case of duryldihydroborane, we were unable to isolate a complex analogous to **2 a** from the reaction with [(Me_3_Si)_2_NBH_2_] (see the Supporting Information for **3 b**, a complex analogous to **3 a**).

Promisingly, these reactions demonstrate that the use of bulky and/or electron‐rich dihydroboranes can lead to full dehydrogenation of dihydroboranes and borylene formation. However, no signs of B−B bond formation were observed. In order to perturb the system further, we turned to an aryldihydroborane bearing an electron‐poor aryl group, namely [3,5‐(CF_3_)_2_C_6_H_3_BH_2_]. Although this unstable borane requires in situ generation and use, its combination with [(Cp*Ru)_2_(μ‐H)_4_] provided a 15 % yield of orange crystals that were determined to consist of an anionic diruthenium complex containing a tetraarylated U‐shaped B_4_ unit, namely Li(THF)_4_[(Cp*Ru)_2_(μ‐B_4_H_5_{3,5‐(CF_3_)_2_C_6_H_3_}_4_)] (**4**), a result of incomplete threefold dehydrocoupling (Scheme 2). The ^11^B NMR spectrum of **4** showed two broad signals at *δ*=−31.1 and 28.7 ppm, and three signals were observed in the upfield region of its ^1^H NMR spectrum (*δ*=−9.64, −8.31, and −6.69) in a 2:2:1 intensity ratio. The solid‐state structure of **4**, as shown in Figure 3, revealed a tetraarylated B_4_ unit stabilized by two ruthenium centers. The B1−B2 (1.709(5) Å) and B3−B4 (1.698(5) Å) distances are significantly shorter than the B2−B3 distance (1.798(6) Å). The Ru−B distances are similar to those of reported ruthenaborane clusters8c, 19 and are in the typical range of single bonds, whereas the two ruthenium centers are too distant from each other (3.676 Å) for there to be any interaction. The core formula of **4** is analogous to Fehlner's *nido*‐[1,2(Cp*Ru)_2_(μ‐H)B_4_H_9_] (**I**),8c and its derivative *nido*‐[1,2(Cp*Ru)_2_(μ‐H)B_4_H_7_Cl_2_],8e which adopt a *nido* pentagonal bipyramidal geometry with one metal atom occupying one of the two axial vertices and the second metal atom and boron atoms the five equatorial vertices. This contrasts with **4**, the core of which has a *nido* geometry based on a pentagonal bipyramidal deltahedron with metal atoms occupying the axial vertices and boron atoms four of the five equatorial vertices. Note that **I** and **4** are isoelectronic (48 cluster valence electrons (cve)) and adopt different isomeric geometries with a metal–metal bond in the former and no metal–metal bond in the latter. It is also worth mentioning that compound **4** strongly resembles Fehlner's 48‐cve diruthenacarborane *nido*‐[1,7‐(Cp*Ru)_2_‐4,5‐Me_2_‐4,5‐C_2_B_2_H_6_].^20^

**Scheme 2:**
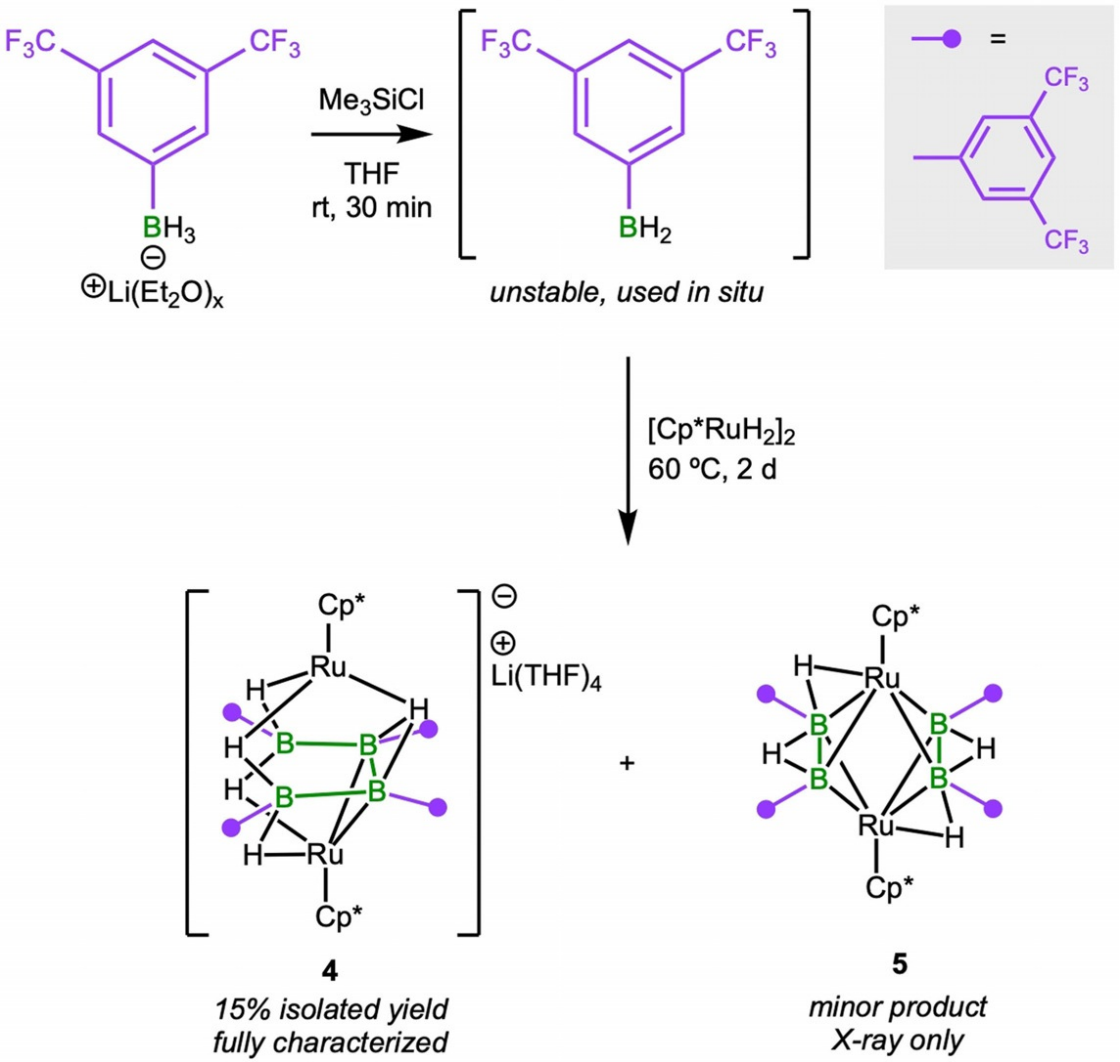
In situ generation and threefold dehydrocoupling of a dihydroborane.

**Figure 3:**
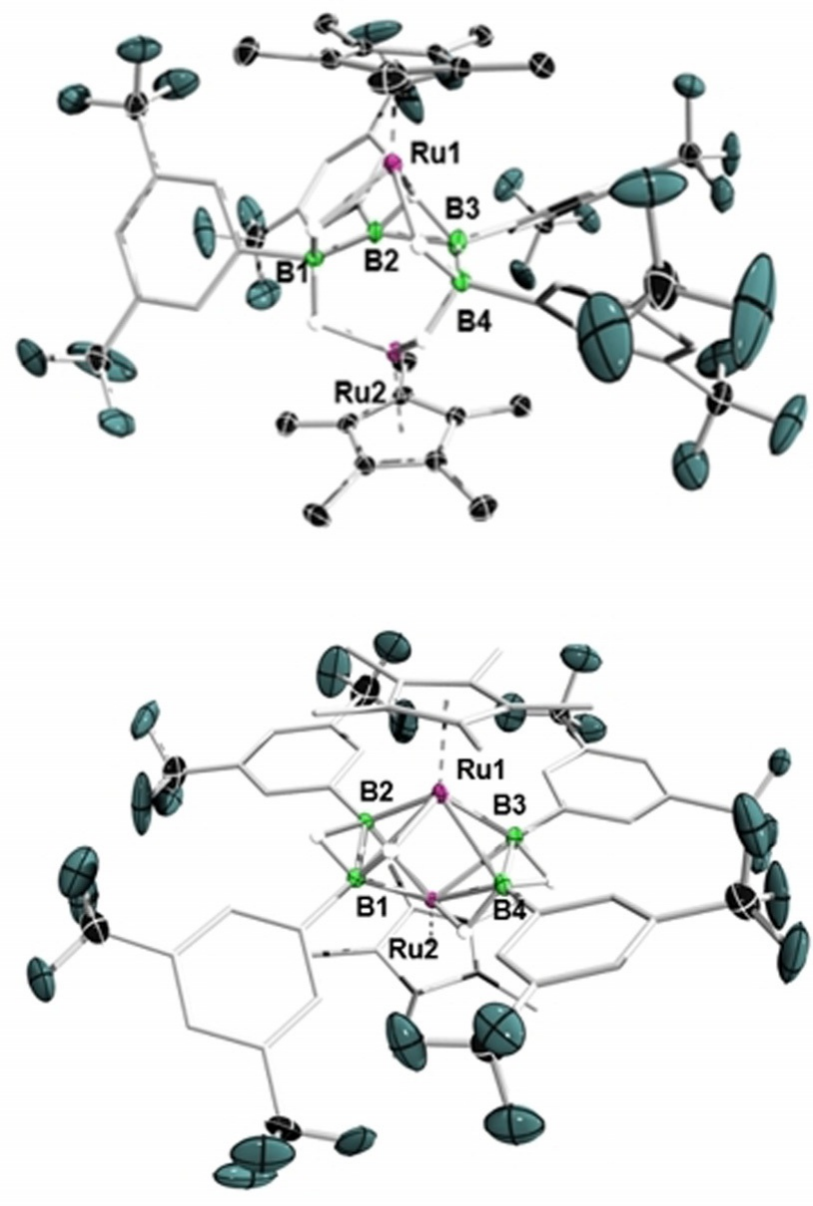
Crystallographically derived structures of **4** (top) and **5** (bottom). Ellipsoids shown at the 50 % probability level. The [Li(THF)_4_]^+^ counterion of **4**, and all hydrogen atoms bound to carbon have been omitted for clarity. Selected bond lengths (Å) and angles (°) for **4**: Ru1−B1 2.360(4), Ru1−B2 2.394(4), Ru2−B3 2.375(4), Ru2−B4 2.365(4), B1−B2 1.709(5), B2−B3 1.798(6), B3−B4 1.698(5); B1‐B2‐B3 112.1(3), B2‐B3‐B4 110.6(3). For **5**: Ru1−B1 2.148(4), Ru1−B2 2.175(4), Ru2−B3 2.171(4), Ru2−B4 2.135(4), B1−B2 1.731(5), B3−B4 1.723(6).

Interestingly, in addition to **4**, a small amount of yellow crystals of a minor product, **5** (see Scheme 2), were also isolated from the reaction mixture. A single‐crystal X‐ray diffraction study revealed that in **5** two diborane(4) units are stabilized by two ruthenium centers, best represented by the formula [(Cp*Ru)_2_(μ‐η^2^:η^2^‐B_2_H_2_{3,5‐(CF_3_)_2_C_6_H_3_}_2_)_2_] (Figure 3). The two B−B units are parallel to each other and perpendicular to the Ru−Ru axis. The B−B bond distances in **5** (1.731(5) and 1.723(6) Å) match those of complexes in which a metal‐ligand fragment binds to the two H atoms of a planar H−B(sp^3^)−B(sp^3^)−H unit, namely those of Kodama and Shimoi^21^ and Himmel.^22^

Density functional theory (DFT) calculations were performed using ADF^23^ at the ZORA‐BLYP‐D3(BJ)/TZ2P level of theory on the model compound [(Cp*Ru)_2_B_4_H_9_]^−^ (**4‐H**), in which the four 3,5‐(CF_3_)_2_C_6_H_3_ units of **4** were replaced with four hydrogen atoms (see the Supporting Information, Figure S23), in order to compare its bonding properties with those of the previously reported related (non‐arylated) compounds [(Cp*_2_Ru_2_)B_4_H_10_] (**I**) (48 cves),8c [(Cp*_2_Cr_2_)B_4_H_8_] (**II**) (42 cves),8d and [(Cp*_2_Re_2_)B_4_H_8_] (**III**) (44 cves), which were also calculated (Figure 4).8e

**Figure 4:**
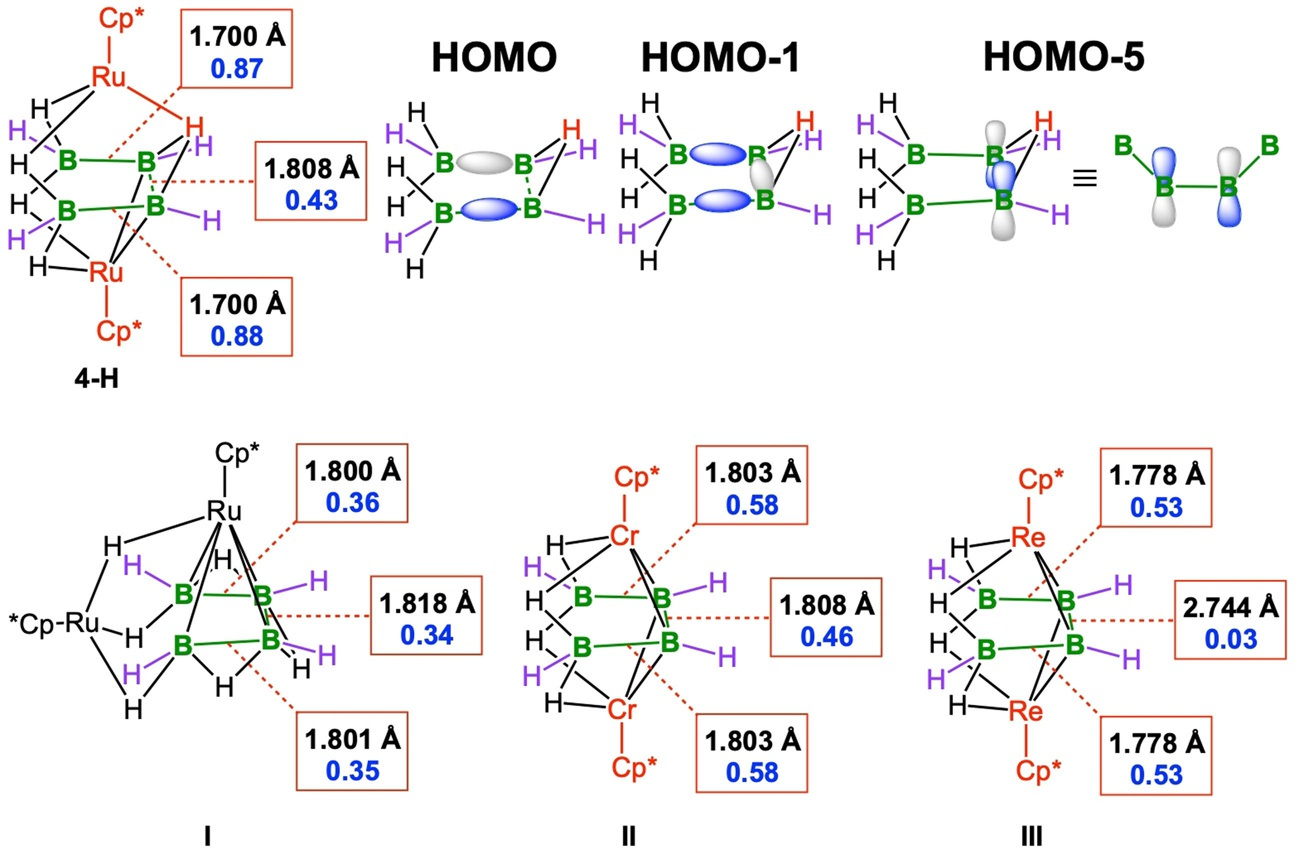
Distances (black type) and MBOs (blue type) of **4‐H** (top left), **I–III** (bottom), and important molecular orbitals of **4‐H** (top right, schematic, only the B_4_H_9_^3−^ subunit is shown).

The calculated B−B distances of **4‐H** and **I** show excellent agreement with the distances obtained experimentally for **4** and **I**, respectively (Table S1, Supporting Information). A HOMO–LUMO gap of 1.58 eV was computed for **4‐H**, indicating that **4** possesses good thermodynamic stability. Interestingly, **4‐H** can be considered to contain two B_2_H_4_^2−^ (=B_4_H_8_^4−^ in total) moieties that are weakly bound to each other, interacting with two [Cp*Ru]^+^ fragments and a proton. B−B σ‐bonds are present in each B_2_H_4_^2−^ unit. Indeed, quantitative Kohn–Sham MO analyses reveal that the HOMO and HOMO−1 calculated for **4‐H** comprise σ‐bonding interactions between the two outer B−B atoms in the B_4_H_8_^4−^ unit (i.e. the B1−B2 and B3−B4 bonds), although their contribution to the total MO is relatively small (19 and 17 %, respectively). The largest contribution to these orbitals is formed by d‐orbitals of the Ru atoms, which interact in an antibonding fashion with the filled B−B σ‐bonds (Figure S26, Supporting Information). In agreement with the presence of B−B σ‐bonds, the Mayer bond orders (MBOs) of the outer (B1−B2 and B3−B4) bonds are close to unity (0.87, 0.88), whereas the internal B−B bond (B2−B3) has an MBO of 0.43. This agrees well with the presence of two B_2_H_4_^2−^ moieties that are connected by a weaker B−B bond. (Figures S25 and S26, Supporting Information). A closer inspection of the frontier orbitals reveals metal‐to‐ligand backbonding (HOMO−5), resulting from donation of electron density from filled metal orbitals to π*(BB) orbitals. The acceptor orbital is formed by the antibonding combination of two p orbitals of B2 and B3 (Figure 4), thereby weakening the interaction between the two B_2_H_4_^2−^ moieties. The resulting B_4_H_8_^4−^ unit interacts with the two [Cp*Ru]^+^ fragments, the single Ru‐bound hydrogen of which bridges the internal BB bond.

The Mayer bond orders and orbitals of the model complex **4‐H** were additionally compared with those arising from a single‐point calculation on complex **4**. The results are displayed in Figure S25 (Supporting Information) and indicate that replacing the four 3,5‐(CF_3_)_2_C_6_H_3_ units of **4** with four hydrogen atoms in **4‐H** has almost no effect on the bonding situation of the subunit. In marked contrast, there are no σ‐bonds between boron atoms in the complexes **I** and **II**. Instead, for complex **I**, HOMO through HOMO−3 are solely formed by non‐bonding metal d‐orbitals. This agrees well with the MBOs of the B−B bonds, which are between 0.34–0.36 (Figure S25, Supporting Information). Interestingly, in the Group 6 complex [(Cp*_2_Cr_2_)B_4_H_8_] (**II**), the LUMO and LUMO+1 correspond to the HOMO and HOMO−1 of complex **4‐H**, that is, they comprise bonding σ interactions between the boron atoms. Consequently, the MBOs are smaller (0.58) and the B−B distances are longer (1.803 Å) than those of the outer B−B bonds in **4‐H**. Similarly, the LUMO and HOMO−1 of Group 7 metal complex **III** ([(Cp*_2_Re_2_)B_4_H_8_]) correspond to the HOMO and HOMO−1 of complex **4‐H**. This is accompanied by a shortening of the terminal B−B bonds from 1.803 (**II**) to 1.778 Å (**III**), whereas the MBOs do not change significantly (0.53). Therefore, only in [(Cp*Ru)_2_B_4_H_9_]^−^ (**4‐H**) are both outer B−B bonds fully formed, giving high B−B bond orders and suggesting the presence of σ‐bonds in each B_2_H_4_^2−^ unit.

We have herein demonstrated the exceptional ability of the diruthenium tetrahydrido complex [(Cp*Ru)_2_(μ‐H)_4_] to mediate the dehydrogenation of functionalized dihydroboranes, leading to bridging borylene complexes in the case of bulky and/or electron‐rich dihydroboranes. However, with the electron‐poor aryldihydroborane [3,5‐(CF_3_)_2_C_6_H_3_BH_2_], we isolated a complex bearing a unit comprising four connected boron atoms, representing the first example of a perarylated boron chain, albeit one with a significantly weaker central B−B bond. Computational results suggest that the bonding in the B_4_ unit of this complex more closely resembles a network of classical σ‐bonds than previously reported clusters with B_4_ networks, thus making it the closest we have come to the construction of a B_4_ chain through B−B dehydrocoupling.

## Conflict of interest

The authors declare no conflict of interest.

## Supporting information

As a service to our authors and readers, this journal provides supporting information supplied by the authors. Such materials are peer reviewed and may be re‐organized for online delivery, but are not copy‐edited or typeset. Technical support issues arising from supporting information (other than missing files) should be addressed to the authors.

Supplementary
