# Supplementary material for: Toward Transition‐Metal‐Templated Construction of Arylated B4 Chains by Dihydroborane Dehydrocoupling
Source: Chemistry. 2019 Dec 9;25(72):16544–9. doi: 10.1002/chem.201904772 (PMC6972581; doi:10.1002/chem.201904772)
Supplement: Supplementary file 1 — Supplementary [file CHEM-25-16544-s001.pdf]

CHEMISTRY
A European Journal

Supporting Information

Toward Transition-Metal-Templated Construction of Arylated B_4 Chains by Dihydroborane Dehydrocoupling

Carsten Lenczyk,[a] Dipak Kumar Roy,[a, b] Kai Oberdorf,[a] Jörn Nitsch,[a] Rian D. Dewhurst,[a] Krzysztof Radacki,[a] Jean-François Halet,*[c] Todd B. Marder,*[a] Matthias Bickelhaupt,*[d] and Holger Braunschweig*[a]

chem_201904772_sm_miscellaneous_information.pdf

Experimental Details

General experimental considerations: All syntheses were carried out in an argon-filled glovebox or with standard Schlenk techniques. All solvents were purified by distillation using appropriate drying agents, deoxygenated using three freeze-pump-thaw cycles and stored over molecular sieves under dry argon prior to use. Deuterated solvents used for NMR spectroscopy were purchased from Cambridge Isotope Laboratories, deoxygenated by freeze-pump-thaw cycles and stored over molecular sieves under dry argon prior to use. All solution NMR spectra were acquired at ambient temperature on a Bruker Avance 400 NMR spectrometer (^{1}H: 400.1 MHz, ^{11}B NMR 128.4 MHz, ^{13}C: 101 MHz, ^{19}F: 376.5 MHz) or a Bruker Avance I 500 spectrometer (^{1}H: 500.1 MHz, ^{11}B NMR 160.5 MHz, ^{13}C: 125.8 MHz). ^{1}H NMR spectra were referenced via residual proton resonances of C_6D_6 (7.16 ppm), THF-d_8 (3.58, 1.72 ppm) and ^{13}C{^{1}H} spectra were referenced to C_6D_6 (128.06 ppm) or THF-d_8 (67.21, 25.31 ppm). High-resolution mass spectrometry was obtained from a Thermo Scientific Exactive Plus spectrometer either in LIFDI or ESI mode. Cp*H,[1] [Cp*RuCl$_2$]$_2$,[2] [Cp*RuH$_2$]$_2$,[3] [DurBH$_2$]$_2$,[4] [(Me$_3$Si)$_2$NBH$_2$],[5] and 3,5-Li[H$_3$B{3,5-C$_6$H$_3$(CF$_3$)$_2$}][6] were prepared as described previously. Me$_3$SiCl was obtained from commercial sources and purified by distillation under an argon atmosphere.

Synthesis of [(Cp*RuH)$_2$BDur] (1a)

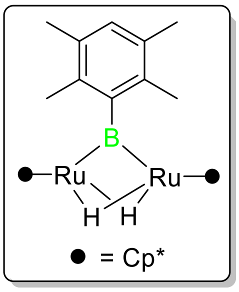

[Cp*RuH$_2$]$_2$ (100 mg, 0.21 mmol) and DurBH$_2$ (30 mg, 0.21 mmol) were added to a sealable NMR tube and dissolved in THF (0.5 mL). Immediate gas evolution was observed after shaking the reaction mixture. The tube was heated to 60 °C for 3 d and a color change from red to yellow was observed. The tube was subsequently shaken to redissolve the precipitated starting material. The solvent was removed under reduced pressure and the black reaction mixture was washed with pentane. The product [(Cp*RuH)$_2$BDur] was obtained by rinsing the remaining black solid with benzene. Slow evaporation of the solution led to yellow crystals suitable for X-ray analysis (Yield: 15 mg, 0.024 mmol, 11%).

^{1}H NMR (500.1 MHz, C_6D_6, 296 K): δ = –12.28 (s, 2H, Ru-*H*-Ru), 1.68 (s, 30H, Cp*-C*H*$_3$), 2.24 (s, 6H, C*H*$_3$Dur), 2.91 (s, 6H, C*H*$_3$Dur), 6.97 (s, 1H, CH^{para})
^{11}B NMR (160.5 MHz, C_6D_6, 296 K): δ = 127.4 (br s)
^{13}C NMR (125.8 MHz, C_6D_6, 296 K): δ = 11.4 (s, Cp*-*C*H$_3$), 19.3 (s, *C*H$_3$-Dur), 19.6 (s, *C*H$_3$-Dur), 89.5 (s, Cp*-C^q), 130.8 (s, CH^{para}), 133.45 (s, C^q-Dur) 133.8 (s, C^q-Dur).
HRMS (LIFDI): calculated for $C_{30}H_{43}BRu_2$ ($[M-2H]^+$) 618.1546; found 618.1530.

Synthesis of $[(Cp^*RuH)_2BN(SiMe_3)_2]$ (1b)

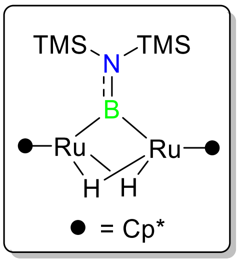

$[Cp^*RuH_2]_2$ (100 mg, 0.21 mmol) and $(Me_3Si)_2NBH_2$ (36 mg, 0.21 mmol) were added to a sealable NMR tube and dissolved in THF (0.5 mL). Immediate gas evolution was observed after shaking the reaction mixture. The tube was heated to 60 °C for 3 d and a color change from red to yellow was observed. The tube was subsequently shaken to redissolve the precipitated starting material. The solvent was removed under reduced pressure and the black reaction mixture was washed with pentane. The product $[(Cp^*RuH)_2BN(SiMe_3)_2]$ was obtained by rinsing the remaining black solid with benzene. Slow evaporation of the solution led to yellow crystals suitable for X-ray analysis (yield: 13 mg, 0.02 mmol, 10%). Dissolving the crystals led to a small amount of decomposition due to the evolution of Me_3SiH detected in the ^1H NMR spectra.

<u>^1H NMR (500.1 MHz, C_6D_6, 296 K):</u> δ = –13.17 (s, 2H, Ru-*H*-Ru), 0.56 (s, 18H, C*H*$_3$-TMS), 1.90 (s, 30H, Cp*-C*H*$_3$)
<u>^{11}B NMR (160.5 MHz, C_6D_6, 296 K):</u> δ = 91.5 (br s)
<u>^{13}C NMR (125.8 MHz, C_6D_6, 296 K):</u> δ = 4.4 (s, *C*H$_3$-TMS), 12.4 (s, Cp**C*H$_3$), 87.4 (s, Cp*-C^q)
HRMS (LIFDI): calculated for $C_{26}H_{50}BNRu_2Si_2$ ($[M]^+$): 647.1661; found 647.1647.

Synthesis of [Cp*Ru{κ^3-H,H,H-(H_3BDur)}] (3a)

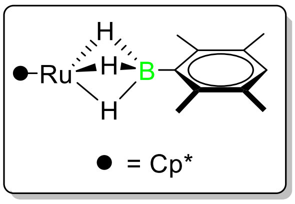

and **[(Cp*Ru)$_2$(μ-H)(μ-κ^3-H,H,H-H_3BDur)] (2a)**

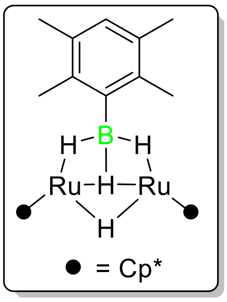

DurBH_2 (60 mg, 0.42 mmol) was added to a stirring THF solution of [Cp*RuH_2]$_2$ (100 mg, 0.21 mmol). An immediate gas evolution was observed. After stirring for 30 min all volatiles were removed in vacuo and the deep-red reaction mixture was dissolved in pentane. Slow evaporation of the solvent provided yellow crystals of [Cp*Ru{κ^3-H,H,H-(H_3BDur)}], which were mechanically separated. Along with this we also separated a few crystals of [(Cp*Ru)$_2$(μ-H){μ-κ^3-H,H,H-(H_3BDur)}]. After washing with cold pentane, the crystals were dried under vacuum and used for characterization.

For [Cp*Ru{κ^3-H,H,H-(H_3BDur)}]:

<u>^{1}H NMR (500.1 MHz, C_6D_6, 296 K):</u> δ = –9.04 (br. s, 3H, Ru-*H*), 1.96 (s, 15H, Cp*-CH_3), 2.06 (s, 6H, Dur-CH_3), 2.49 (s, 6H, Dur-CH_3), 6.86 (s, 1H, CH^{para})

<u>^{11}B NMR (160.5 MHz, C_6D_6, 296 K):</u> δ = 25.4 (br. s)

<u>^{13}C NMR (125.8 MHz, C_6D_6, 296 K):</u> δ = 12.2 (s, Cp*-*C*H_3), 19.2 (s, *C*H_3-Dur), 20.3 (s, *C*H_3-Dur), 84.6 (s, Cp*-*C*q), 132.8 (s, CH^{para}), 139.0 (s, Cq, *C*q-Dur)

For [(Cp*Ru)$_2$(μ-H){μ-κ^3-H,H,H-(H_3BDur)}]:

<u>^{1}H NMR (500.1 MHz, C_6D_6, 296 K):</u> δ = –8.76 (br. s, 4H, Ru-*H*-Ru), 1.81 (s, 30H, Cp*-CH_3), 2.28 (s, 6H, Dur-CH_3), 2.35 (s, 6H, Dur-CH_3), 6.95 (s, 1H, CH^{para})

<u>^{11}B NMR (160.5 MHz, C_6D_6, 296 K):</u> δ = 62.3 (br. s)

Synthesis of [Cp*Ru{κ^3-H,H,H-($H_3B(SiMe_3)_2$)}] (3b)

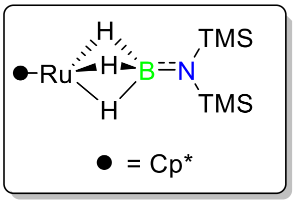

$(Me_3Si)_2NBH_2$ (75 mg, 0.42 mmol) was added to a stirred THF solution of $[Cp^*RuH_2]_2$ (100 mg, 0.21 mmol). An immediate gas evolution was observed. After stirring for 30 minutes all volatiles were removed by vacuum and the deep-red reaction mixture was dissolved in pentane. Slow evaporation of the solvent provided yellow crystals, which were mechanically separated. After washing with cold pentane, the crystals were dried under vacuum. The complex decomposes with liberation of TMS-H upon solvation in C_6D_6.

<u>^{1}H NMR (500.1 MHz, C_6D_6, 296 K):</u> δ = –10.38 (br. s, 3H, Ru-*H*), 0.23 (s, 18H, C*H*$_3$-TMS), 2.01 (s, 15H, Cp*-C*H*$_3$)

<u>^{11}B NMR (160.5 MHz, C_6D_6, 296 K):</u> δ = 31.5 (br. s)

<u>^{13}C NMR (125.8 MHz, C_6D_6, 296 K):</u> δ = 1.0 (s, *C*H$_3$-TMS), 11.9 (s, Cp*-*C*H$_3$), 85.8 (s, Cp*-*C*q)

Synthesis of [Li(THF)$_4$][(Cp*Ru)$_2$B$_4$H$_5${3,5-C$_6$H$_3$(CF$_3$)$_2$}$_4$] (4)

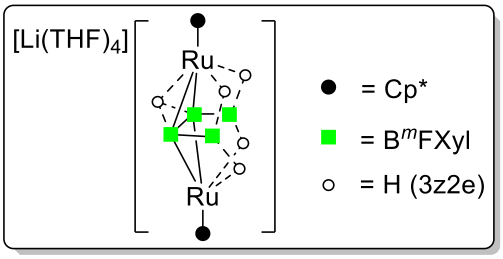

Li[H$_3$B{3,5-C$_6$H$_3$(CF$_3$)$_2$}].2(Et$_2$O) (382.12 mg, 1 mmol, 5 equiv) was dissolved in THF. Me$_3$SiCl (2 mL, 1 molL^{-1}, 10 equiv) was added to the solution at room temperature, causing the clear solution to turn into a white suspension. After 20 min, all volatiles were quickly removed (vacuum was applied to the remaining oil for a further 5 min to ensure no residues of Me$_3$SiCl remained). The oil was then extracted with THF and added to a flask containing [Cp*RuH$_2$]$_2$ (100 mg, 0.2 mmol, 1 equiv). The flask was placed in an oil bath at 60 °C for 2 d. All volatiles were removed in vacuo and the remaining red oil was dissolved in benzene and filtered through a pad of Celite to remove remaining LiCl. After removing the solvent under vacuum, pentane was added to the reaction mixture and, after 5 min, the precipitation of a red solid was observed as well as the formation of red crystals suitable for X-ray crystallography. For characterization, crystals were mechanically picked, washed with pentane (3 x 2 mL) and dried under vacuum to obtain pure material (yield: 51 mg, 15%).

Note: From the above reaction compound **5** was recrystallized as a minor product. This was only characterized by X-ray structure analysis.

<u>^{1}H NMR (500.1 MHz, d$_8$-THF, 296 K):</u> δ = –9.64 (br s, 2H, B-*H*-Ru), –8.31 (br s, 2H, B-*H*-Ru), –6.69 (br s, 1H, B-*H*-B), 1.53 (s, 30H, Cp*-C*H*$_3$), 1.69 (m, 16H, C*H*$_2$-THF), 1.73 (d$_8$-THF), 3.54 (m, 16H, C*H*$_2$-THF), 3.58 (d$_8$-THF), 7.34 (s, 2H, C*H*^{para}), 7.39 (s, 2H, C*H*^{para}), 7.44 (s, 4H, C*H*^{para}), 7.63 (s, 4H, C*H*^{ortho})

<u>^{11}B{1H} NMR (160.5 MHz, d$_8$-THF, 296 K):</u> δ = –31.1 (br), 28.7 (br)

<u>^{13}C NMR (125.8 MHz, d$_8$-THF, 296 K):</u> δ = 9.9 (s, Cp*-*C*H$_3$), 25.1 (s, THF), 67.0 (s, THF), 115.0 (s, *C*H^{para}), 115.8 (s, *C*H^{para}), 124.38 (d, $^1J_{CF}$ = 271 Hz, *C*F$_3$^{meta}) 124.64 (d, $^1J_{CF}$ = 272 Hz, *C*F$_3$^{meta}), 125.7 (s, C^q), 127.7 (d, $^2J_{CF}$ = 31 Hz), 127.7 (d, $^2J_{CF}$ = 31 Hz), 136.3 (s, *C*H^{ortho}), 137.0 (s, *C*H^{ortho}); resonances for *C*^{ipso} and *C*^{q} of Cp* not detected

<u>^{19}F NMR (125.8 MHz, d$_8$-THF, 296 K):</u> δ = –63.2, –63.5

HRMS: ESI (toluene) negative ion mode: m/z calculated for C$_{52}$H$_{47}$B$_4$F$_{24}$Ru$_2$ (M$^-$ without [Li(THF)$_4$]$^+$) 1374.1765, found: 1374.1786 (M$^-$)

NMR Spectra

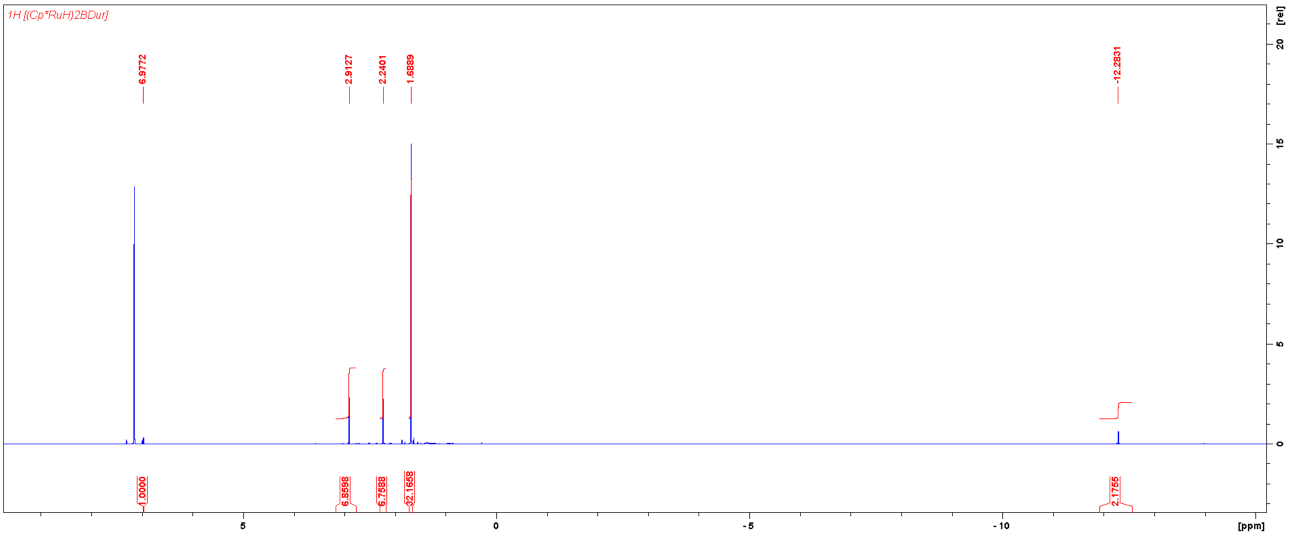

Figure S1. ^{1}H NMR spectrum of [(Cp*RuH)$_2$BDur] (**1a**) in C_6D_6

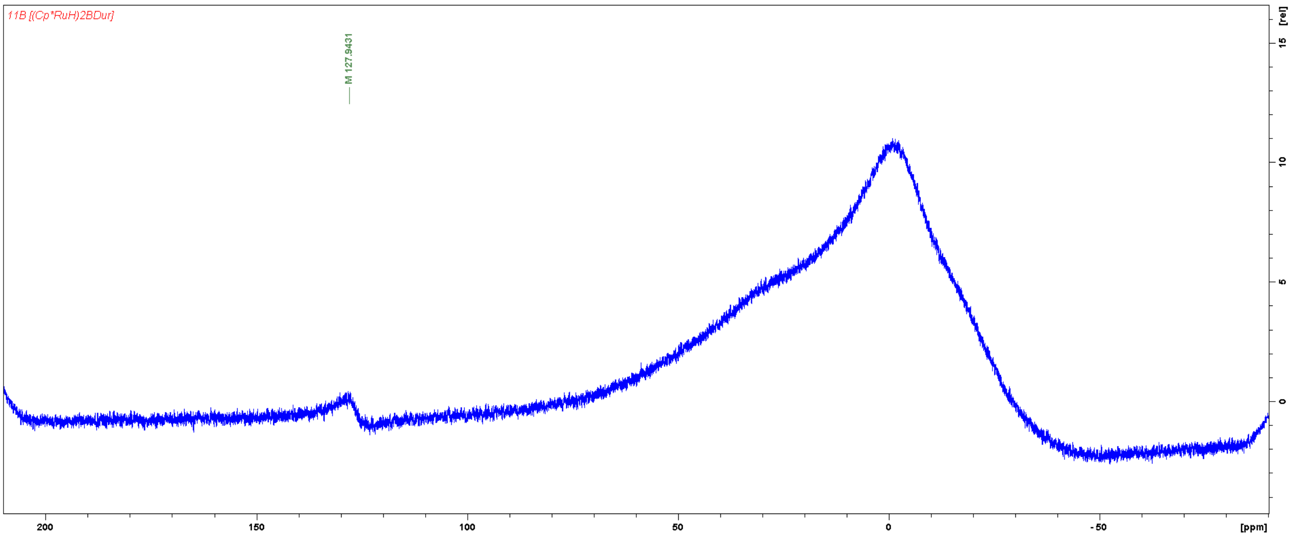

Figure S2. ^{11}B NMR spectrum of [(Cp*RuH)$_2$BDur] (**1a**) in C_6D_6

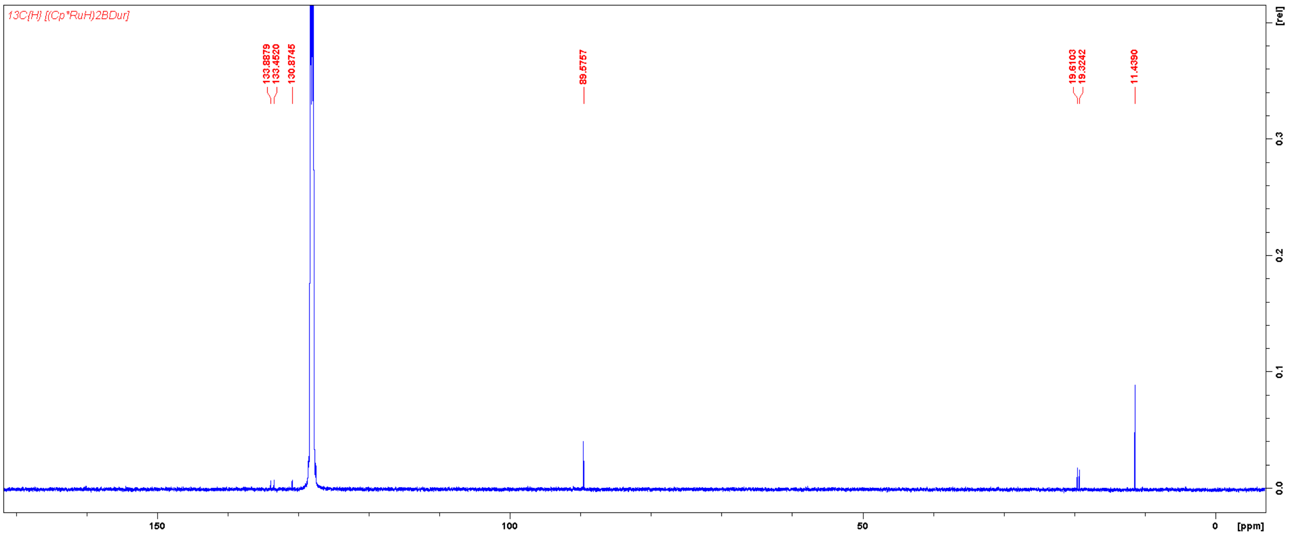

Figure S3. $^{13}C\{^{1}H\}$ NMR spectrum of [(Cp*RuH)$_2$BDur] (**1a**) in C_6D_6

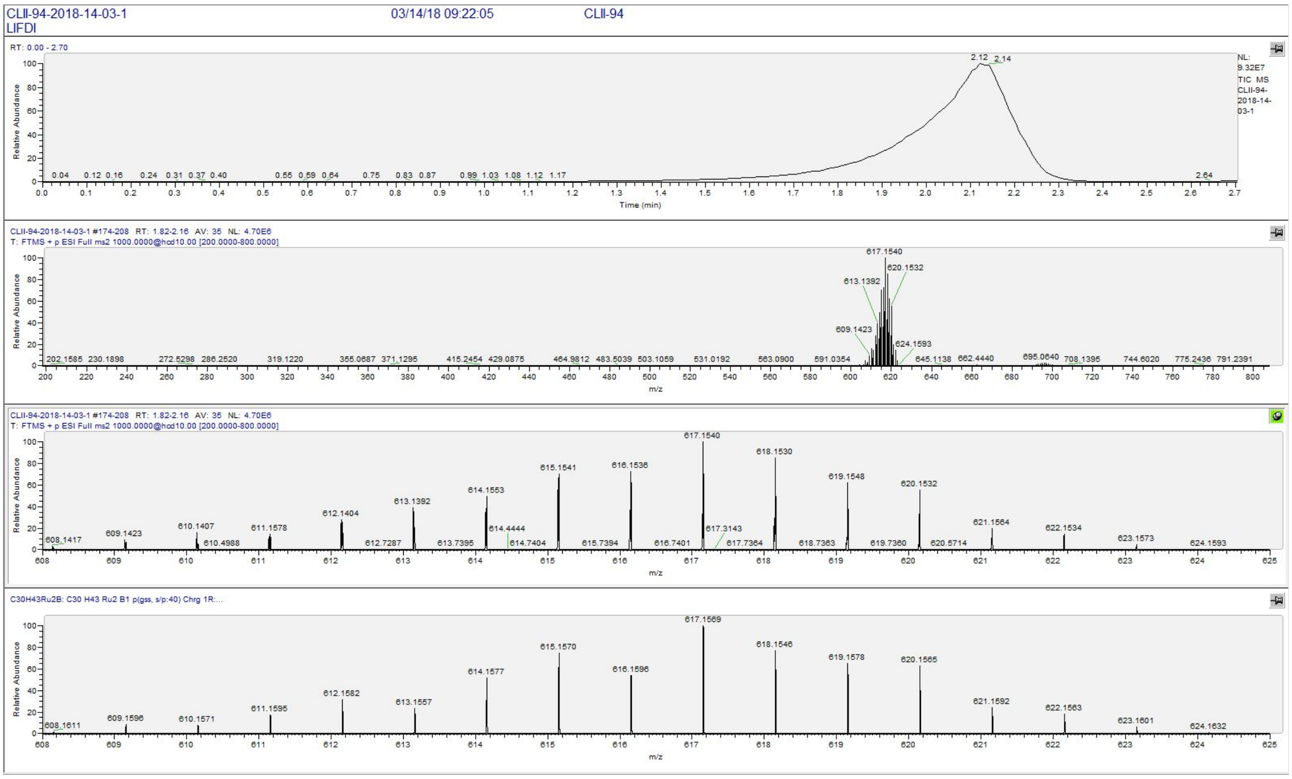

Figure S4. High-resolution mass spectrum (LIFDI) of [(Cp*RuH)$_2$BDur] (**1a**).

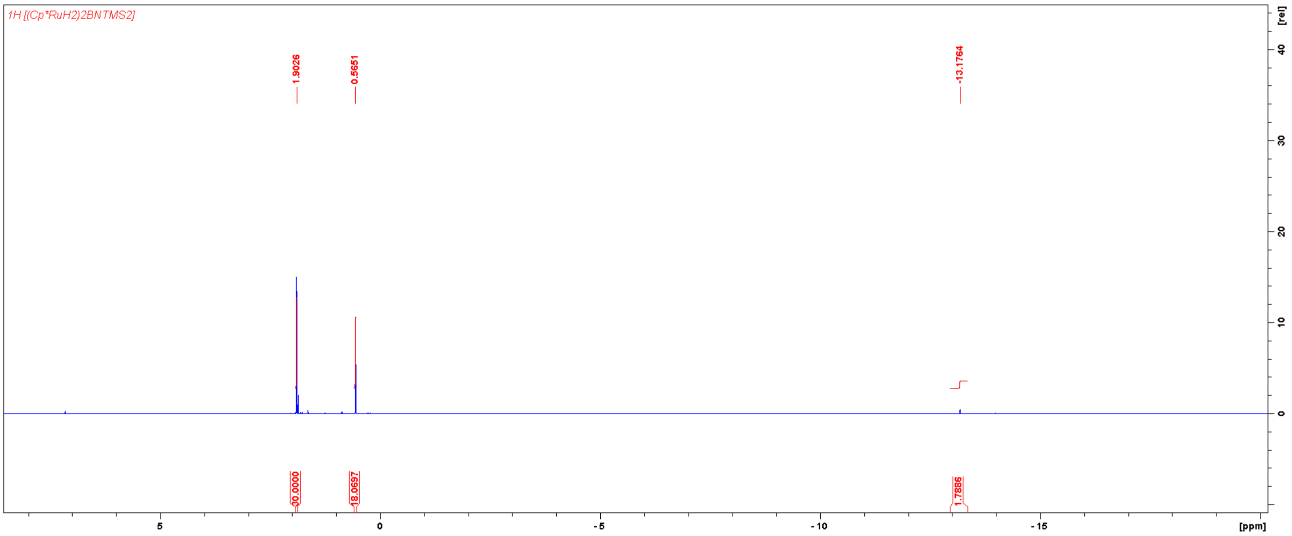

Figure S5. ^{1}H NMR spectrum of $[(Cp^{*}RuH)_2BN(SiMe_3)_2]$ (**1b**) in C_6D_6

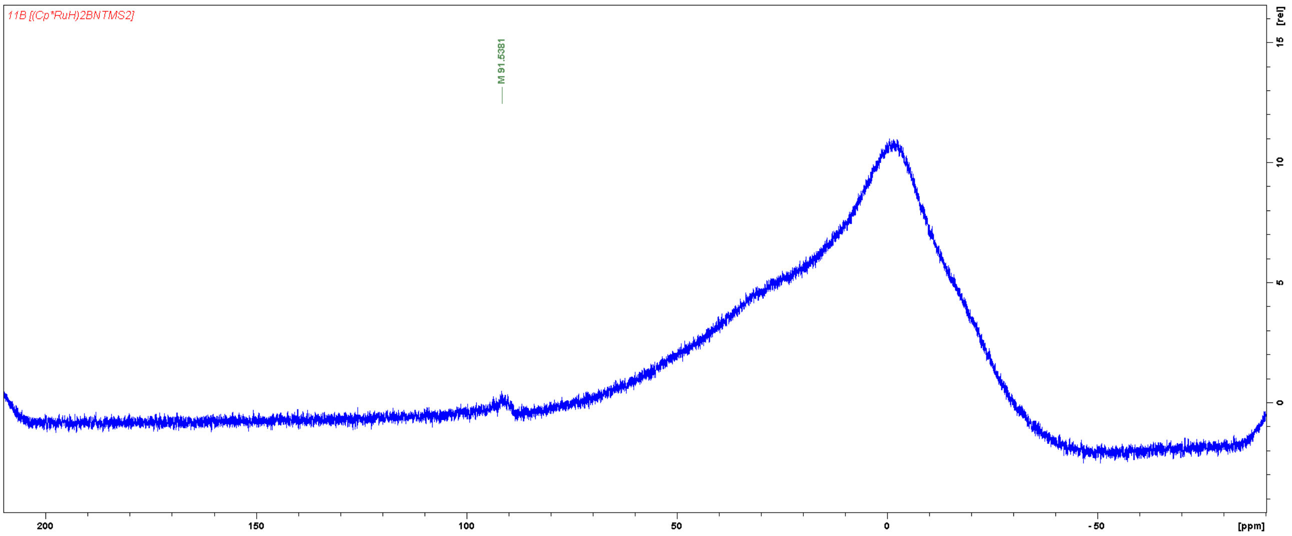

Figure S6. ^{11}B NMR spectrum of $[(Cp^{*}RuH)_2BN(SiMe_3)_2]$ (**1b**) in C_6D_6

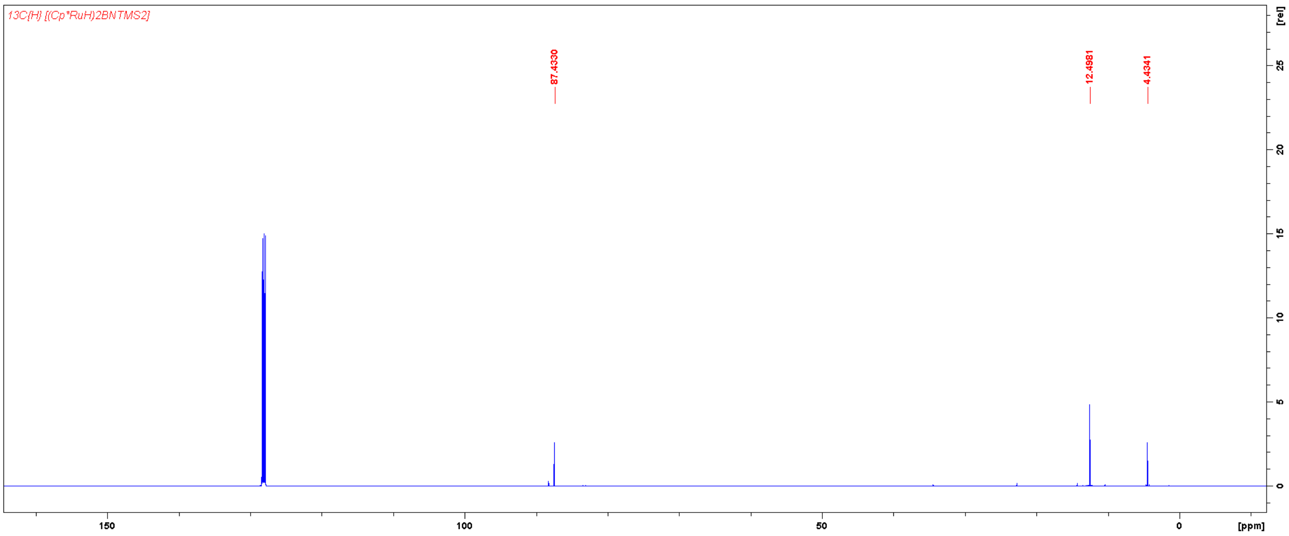

Figure S7. $^{13}C\{^{1}H\}$ NMR spectrum of $[(Cp^*RuH)_2BN(SiMe_3)_2]$ (**1b**) in C_6D_6

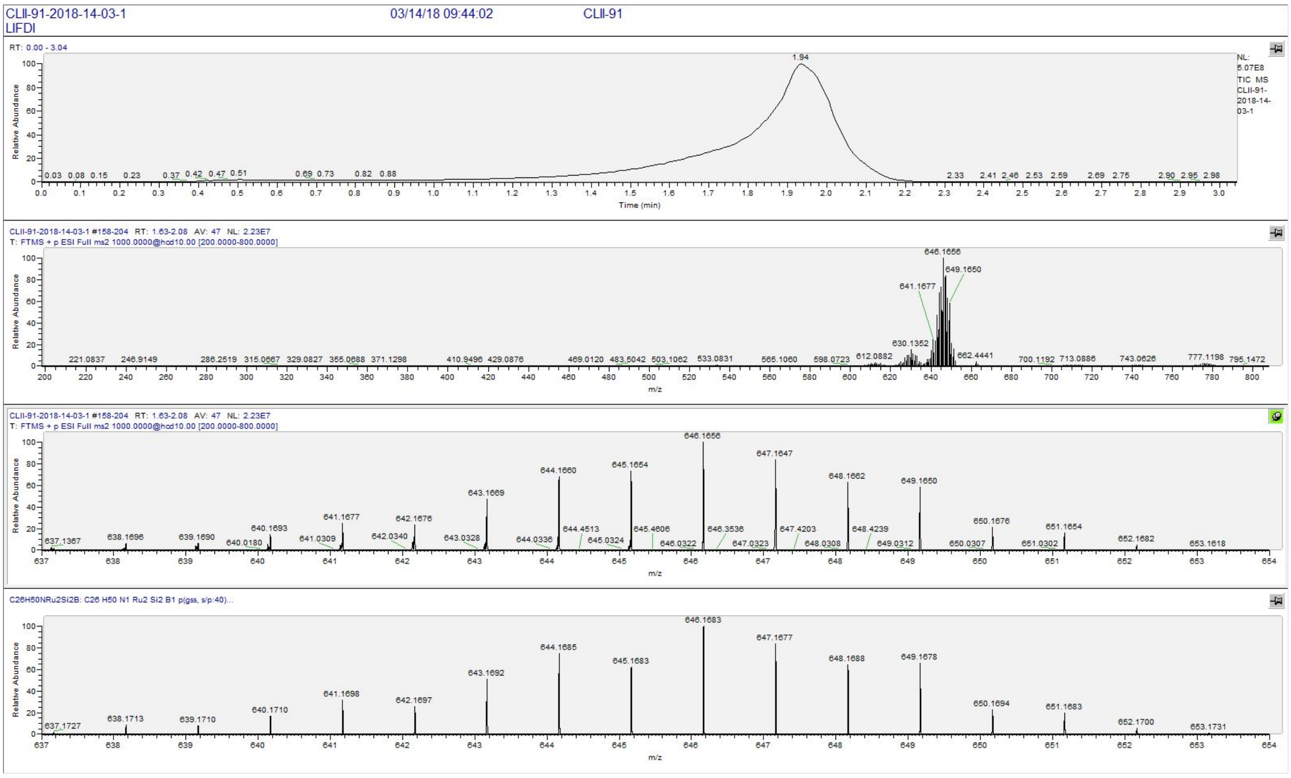

Figure S8. High-resolution mass spectrum (LIFDI) of $[(Cp^*RuH)_2BN(SiMe_3)_2]$ (**1b**).

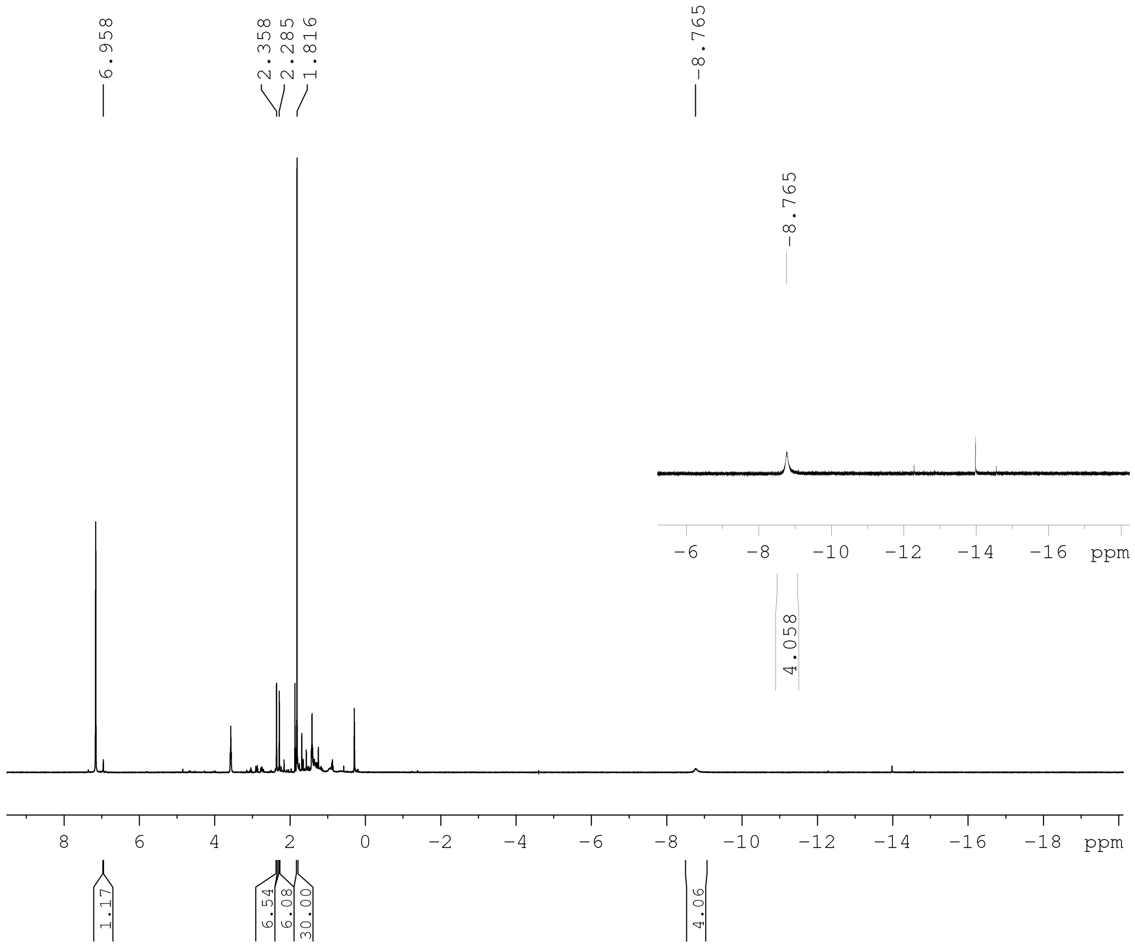

Figure S9. ^{1}H NMR spectrum of [(Cp*Ru)$_2$(μ-H){μ-κ^3-H,H,H-(H_3BDur)}] (**2a**) in C_6D_6

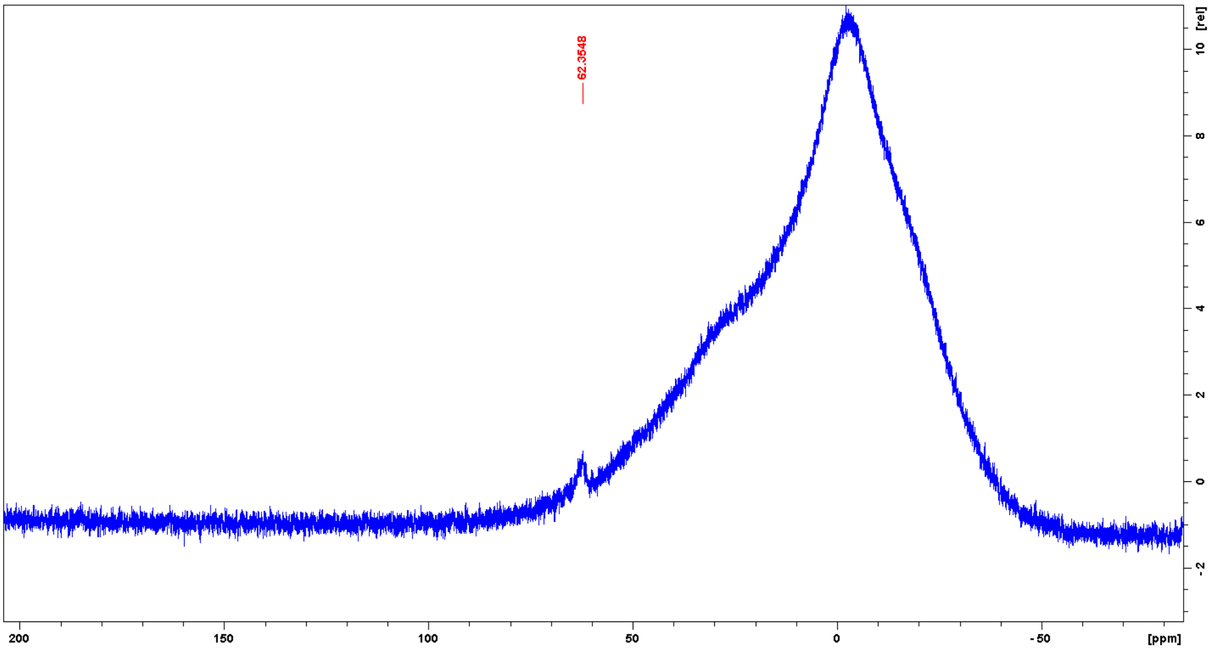

Figure S10. ^{11}B NMR spectrum of [(Cp*Ru)$_2$(μ-H){μ-κ^3-H,H,H-(H_3BDur)}] (**2a**) in C_6D_6

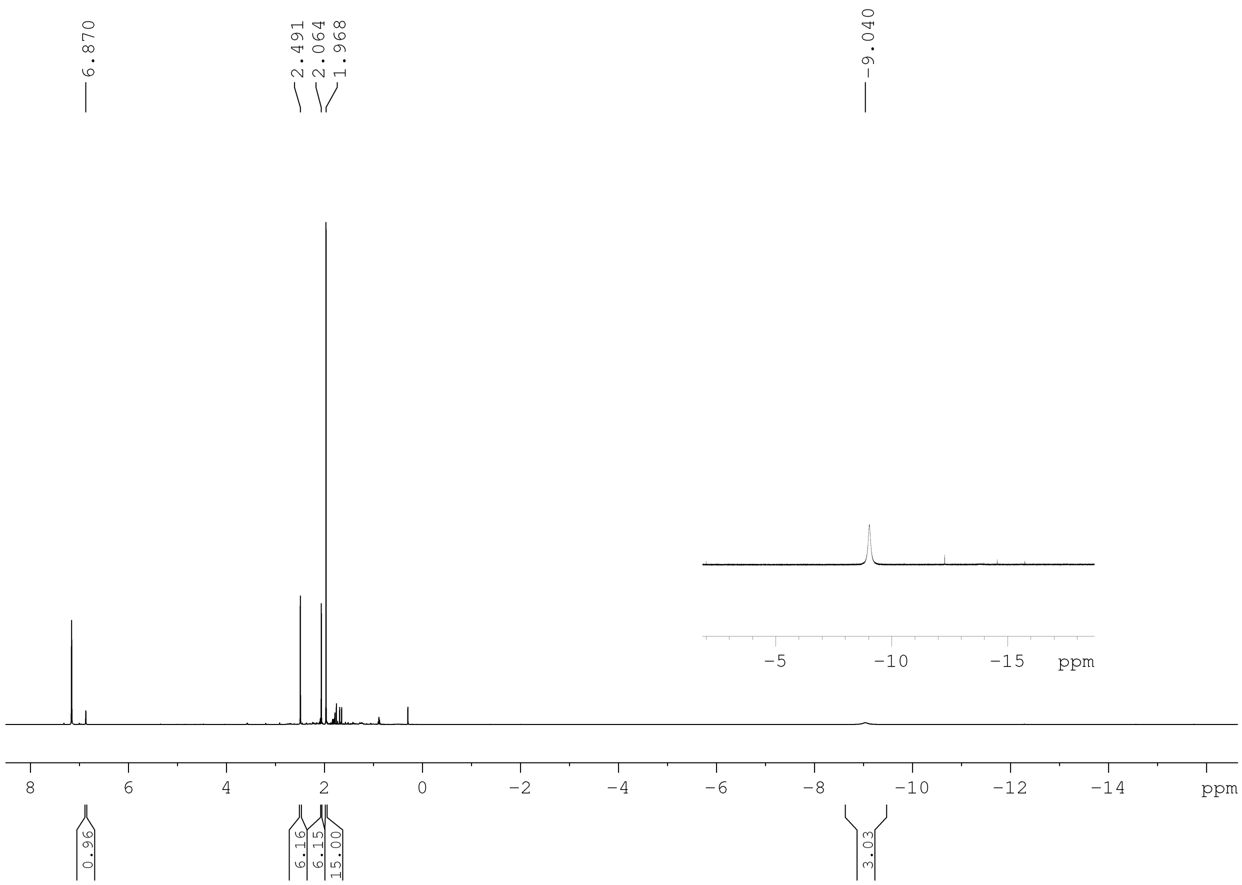

Figure S11. ^{1}H NMR spectrum of [Cp*Ru{κ^3-H,H,H-(H_3BDur)}] (**3a**) in C_6D_6

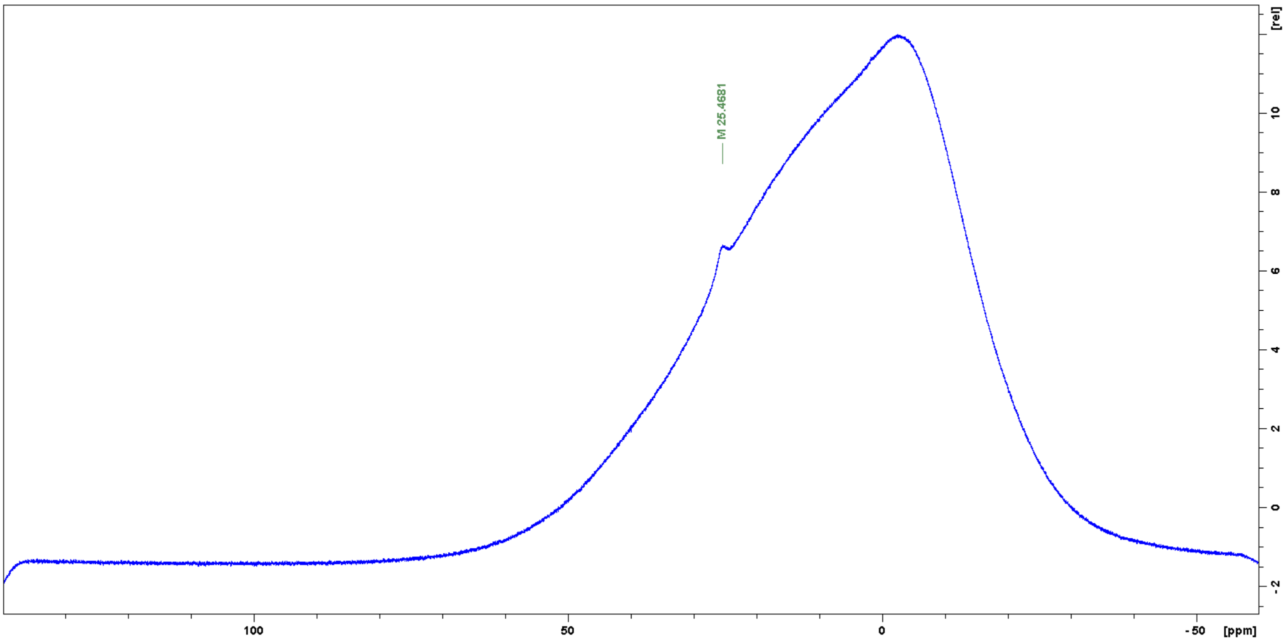

Figure S12. ^{11}B NMR spectrum of [Cp*Ru{κ^3-H,H,H-(H_3BDur)}] (**3a**) in C_6D_6

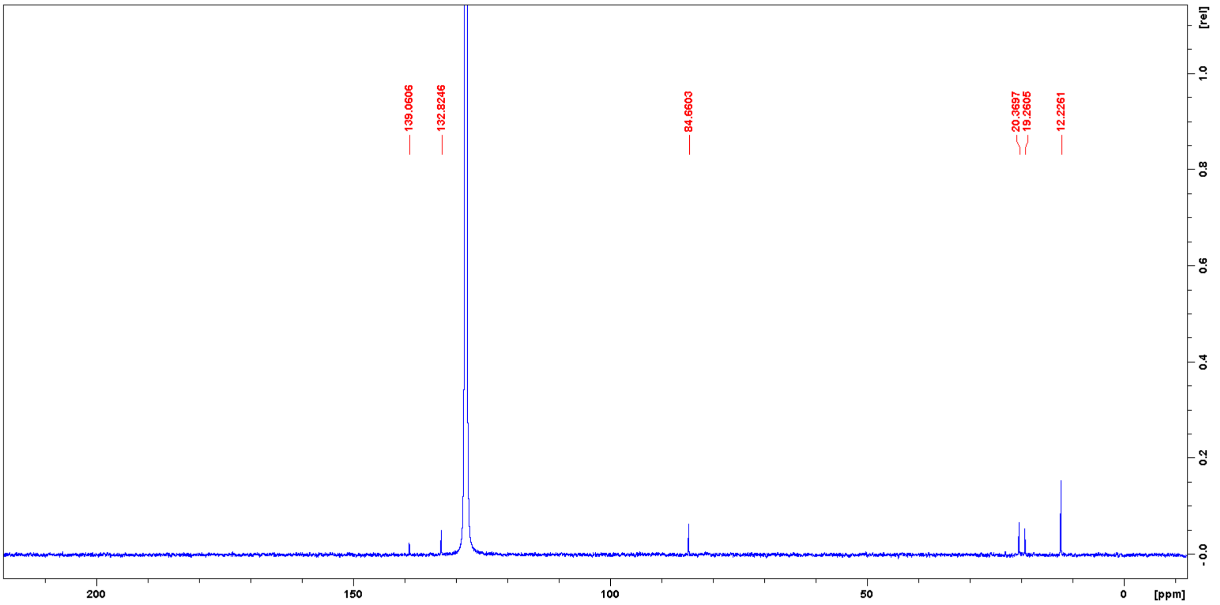

Figure S13. $^{13}C\{^{1}H\}$ NMR spectrum of [Cp*Ru{κ^3-H,H,H-(H_3BDur)}] (**3a**) in C_6D_6

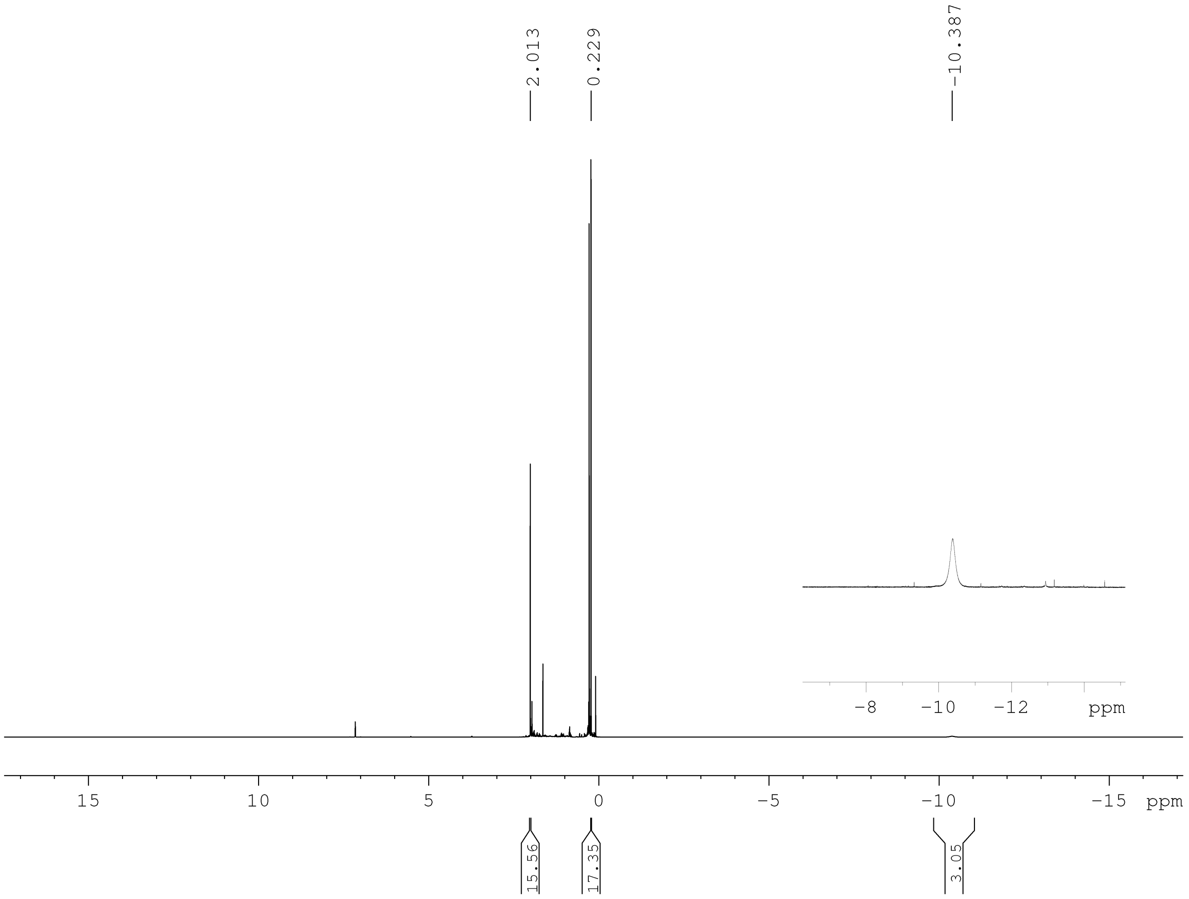

Figure S14. ^{1}H NMR spectrum of [Cp*Ru{κ^3-H,H,H-($H_3BN(SiMe_3)_2$)}] (**3b**) in C_6D_6

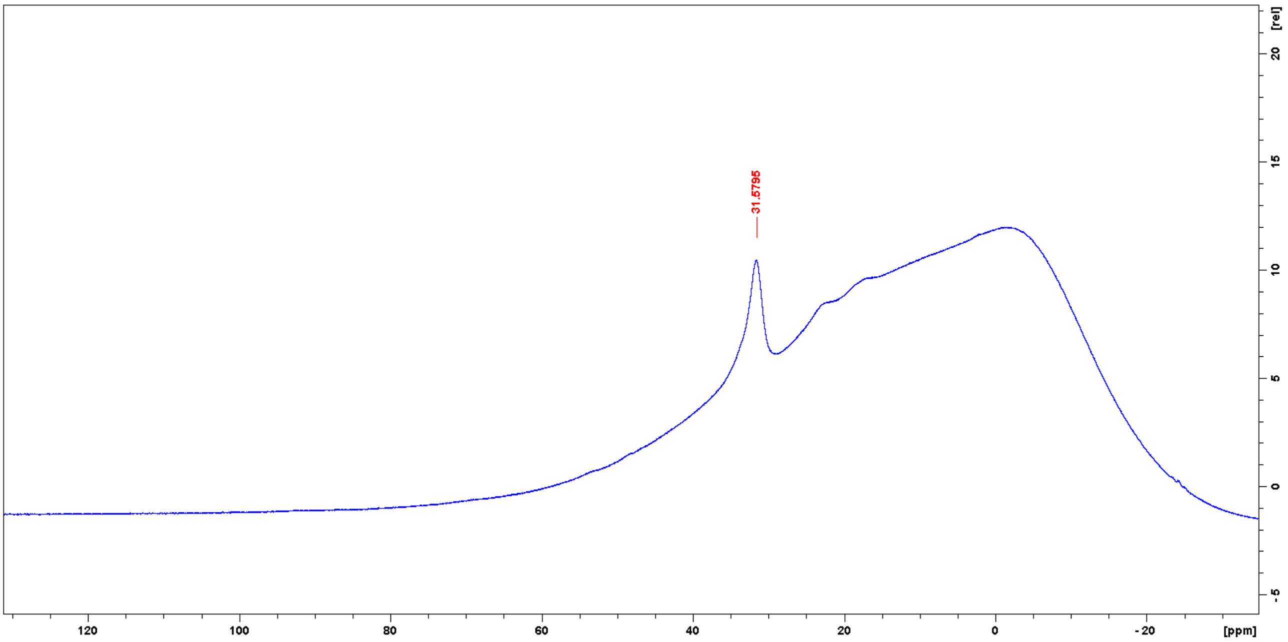

Figure S15. ^{11}B NMR spectrum of [Cp*Ru{κ^3-H,H,H-($H_3BN(SiMe_3)_2$)}] (**3b**) in C_6D_6

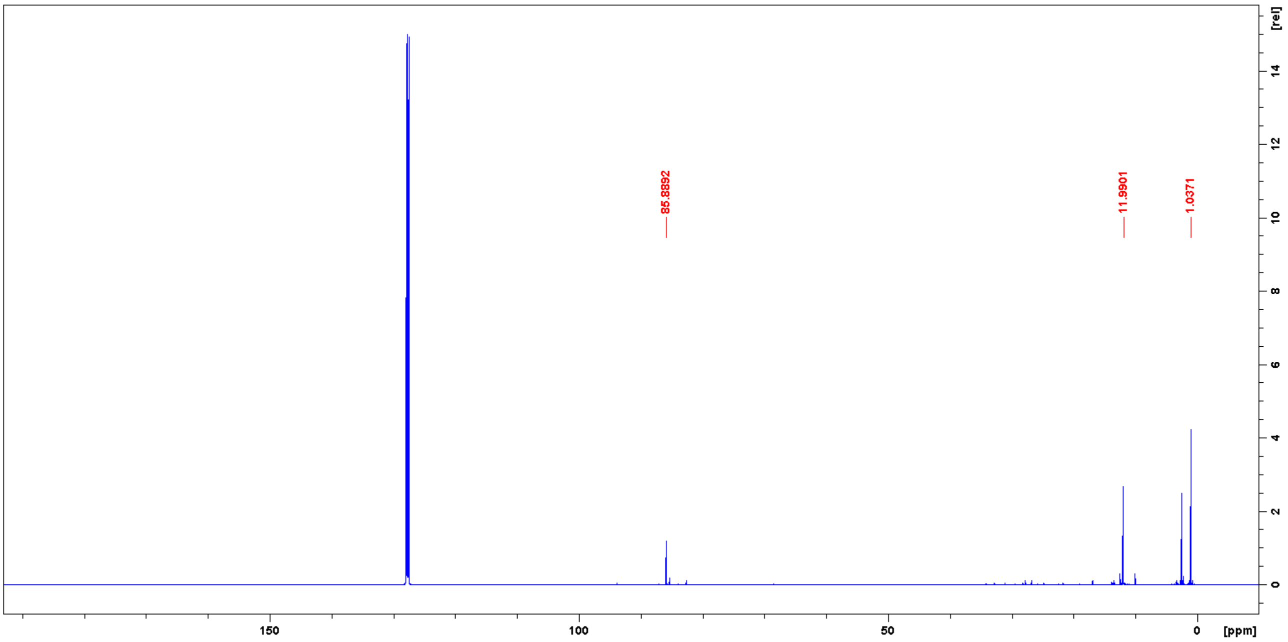

Figure S16. $^{13}C\{^1H\}$ NMR spectrum of [Cp*Ru{κ^3-H,H,H-($H_3BN(SiMe_3)_2$)}] (**3b**) in C_6D_6

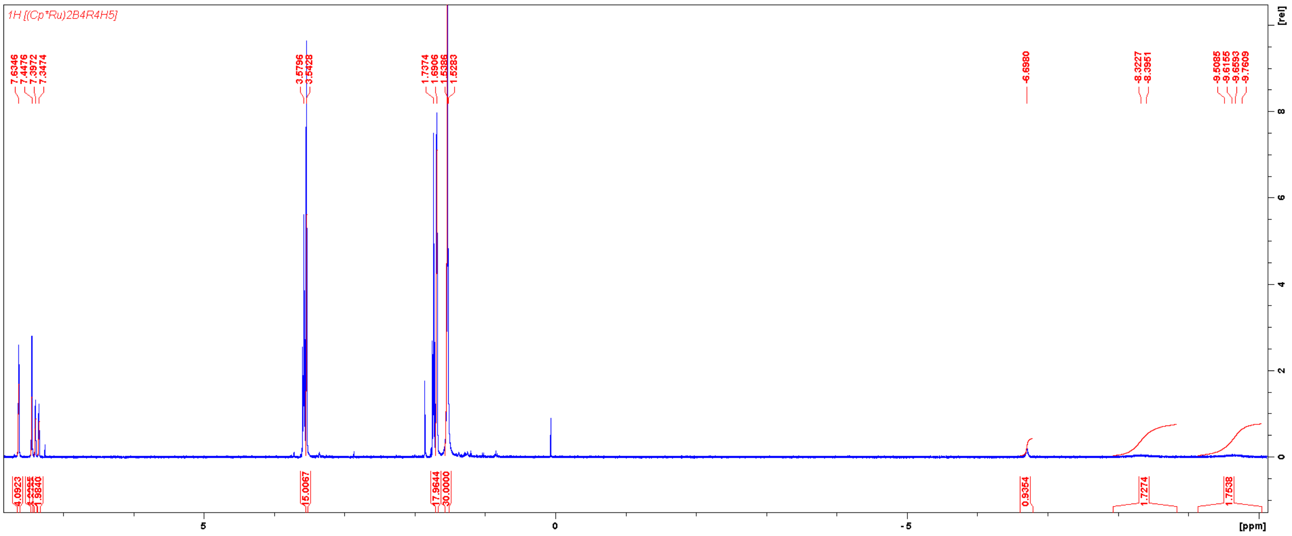

Figure S17. ^{1}H NMR spectrum of $Li[(Cp^{*}Ru)_2B_4H_5\{3,5\text{-}C_6H_3(CF_3)_2\}_4]$ (**4**) in d_8-THF.

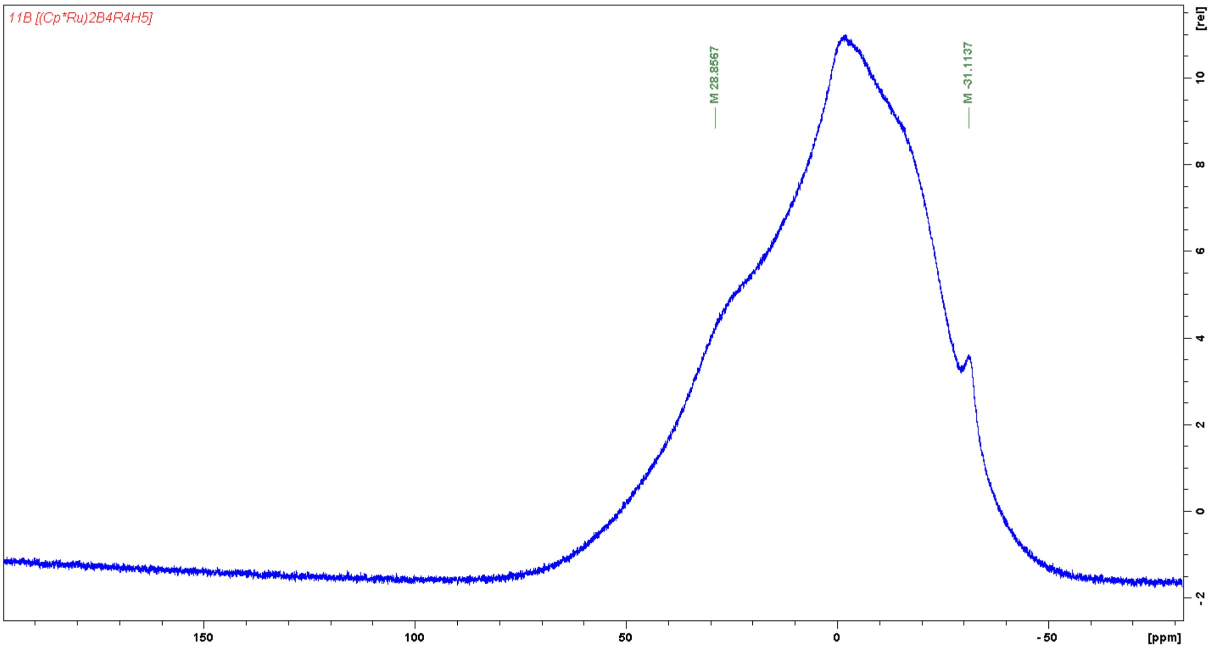

Figure S18. ^{11}B NMR spectrum of $Li[(Cp^{*}Ru)_2B_4H_5\{3,5\text{-}C_6H_3(CF_3)_2\}_4]$ (**4**) in d_8-THF.

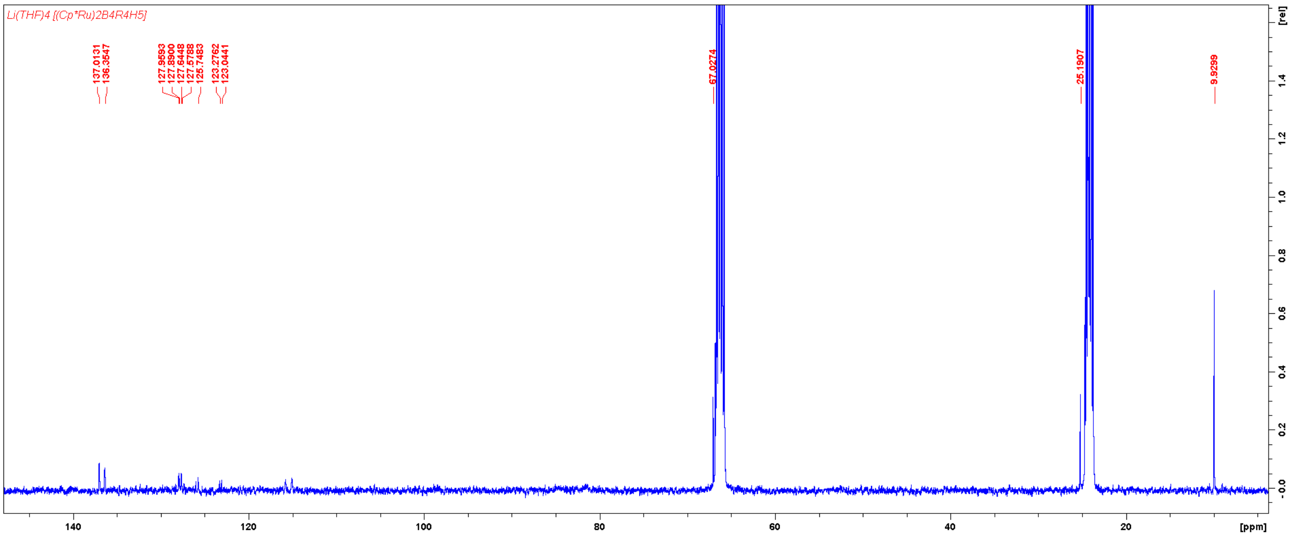

Figure S19. $^{13}C\{^{1}H\}$ NMR spectrum of $Li[(Cp^{*}Ru)_2B_4H_5\{3,5\text{-}C_6H_3(CF_3)_2\}_4]$ (**4**) in d_8-THF.

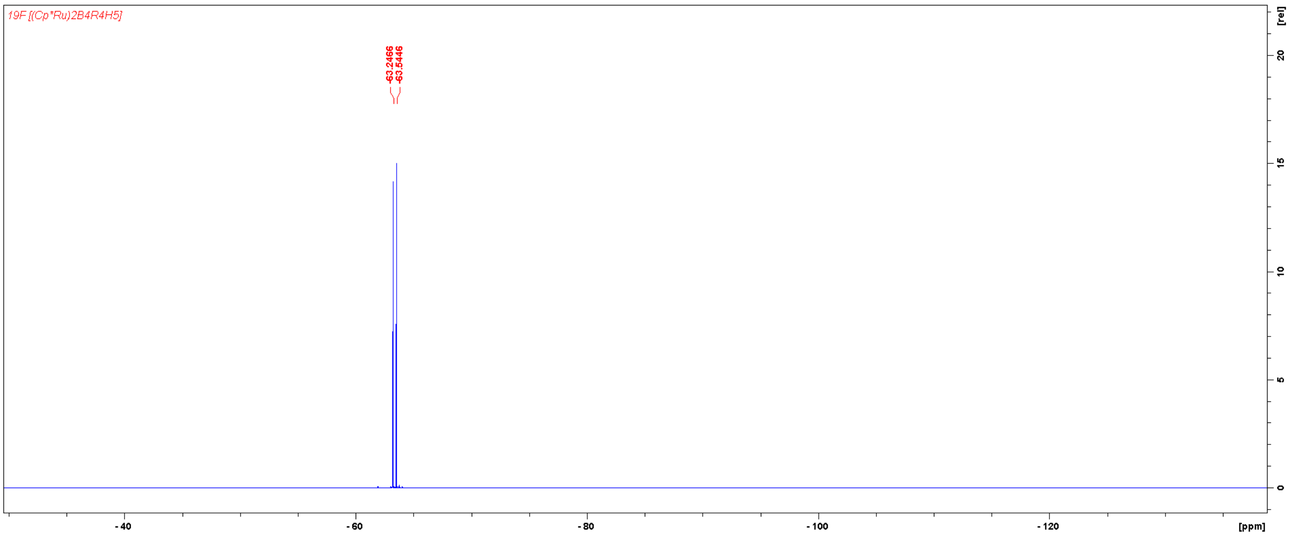

Figure S20. ^{19}F NMR spectrum of $Li[(Cp^{*}Ru)_2B_4H_5\{3,5\text{-}C_6H_3(CF_3)_2\}_4]$ (**4**) in d_8-THF.

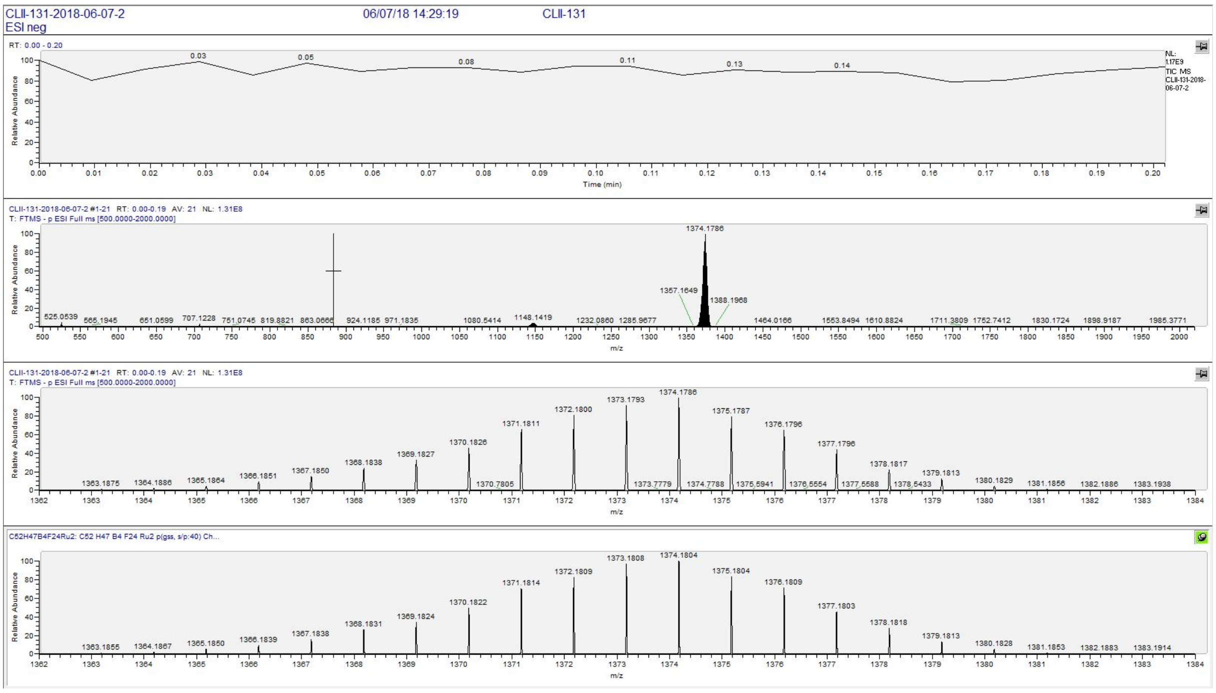

Figure S21. High-resolution mass spectrum (ESI) of Li[(Cp*Ru)$_2$B$_4$H$_5${3,5-C$_6$H$_3$(CF$_3$)$_2$}$_4$] (**4**).

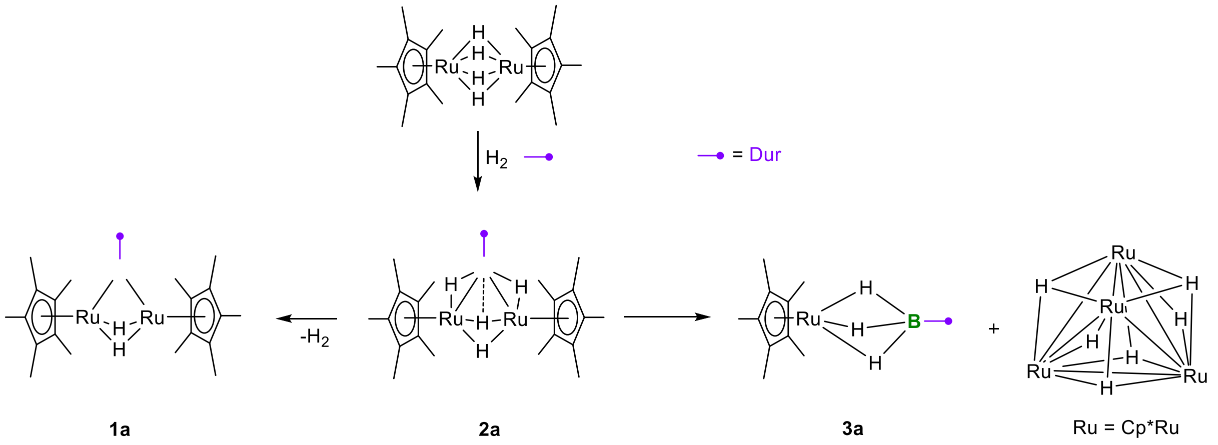

Figure S22. Possible pathway for the formation of bridging borylene complex **1a**.

Density Functional Methods

All calculations were carried out using the Amsterdam Density Functional (ADF) program.[7] The numerical integration was performed by using a procedure developed by Becke *et al.*[8] The molecular orbitals (MOs) were expanded in a large uncontracted set of Slater-type orbitals (STOs) containing diffuse functions: a triple-ζ quality basis set was used for all atoms,[9] augmented with two sets of polarization functions for H (2p, 3d), B, C, F, (3d, 4f) and Ru (5p, 4f). An auxiliary set of s, p, d, f and g STOs was used to fit the molecular density and to represent the Coulomb and exchange potentials accurately in each self-consistent field (SCF) cycle. All electrons were included in the variational treatment (no frozen-core approximation was used). The generalized gradient approximation (GGA) at the BLYP level was used where exchange is described by the Slater Xα potential,[10] with non-local corrections due to Becke[11] added self-consistently, and where correlation was treated by using the Lee-Yang-Parr gradient-corrected functional.[12] Relativistic effects were included with the scalar-zero-order-regular-approximation (ZORA).[13] In addition, the D3(BJ) dispersion correction was used.[14] Energy minima have been verified through vibrational analysis.[15] Mayer bond orders (MBO)[16] and Voronoi deformation density (VDD) charges[17] were calculated for the optimized gas-phase structures at the same level of theory.

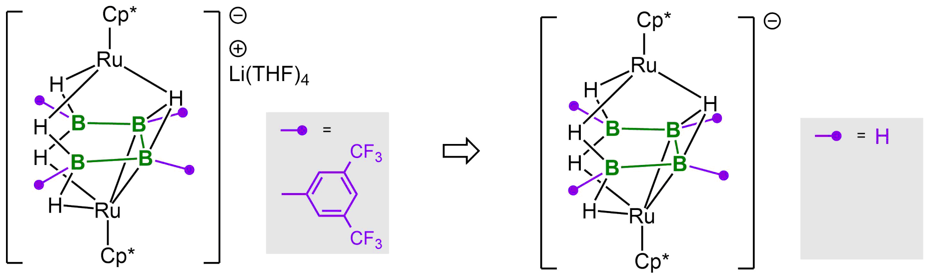

Model compound **4-H**

Figure S23: Compound **4** [(Cp*$_2$Ru$_2$H$_5$)B$_4$Ar$_4$][Li(THF)$_4$] and the truncated model complex **4-H** [(Cp*$_2$Ru$_2$H$_5$)B$_4$H$_4$]$^-$ calculated in this work.

Starting from the geometry obtained from X-ray diffraction, compound **4** was simplified by replacing the four 3,5-(CF$_3$)$_2$-C$_6$H$_3$ groups with four hydrides, leading to model complex **4-H** (Figure 23). We carefully compared our results for the optimized model complex **4-H** with a single point calculation of compound **4** in the geometry obtained from X-ray diffraction. The structural parameters and the calculated Mayer bond orders (MBO) of the BB bonds of the experimentally-obtained complex **4** agree excellently with the calculated values of the model complex **4-H**. These results convinced us that the model system **4-H** is sufficient to describe the bonding situation in the parent complex **4**. The optimzed gas-phase structures of compounds **4-H** and [(Cp*Ru)$_2$(μ-H)B$_4$H$_9$] **I**,[18] [(Cp*$_2$Cr$_2$)B$_4$H$_8$] **II**[19] and [(Cp*$_2$Re$_2$)B$_4$H$_8$] **III**[20] are shown in Figure S24.

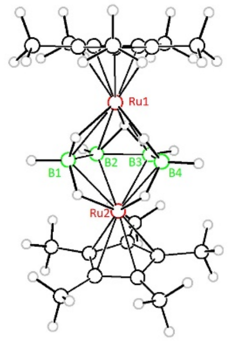

4-H

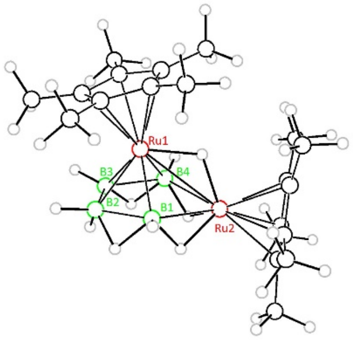

I

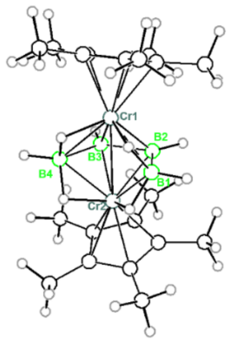

II

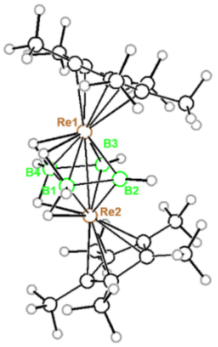

III

Figure S24. Optimized gas-phase structures of the model complex $[(Cp^*{}_2Ru_2)B_4H_9]^-$ (**4-H**) and the literature-known complexes $[(Cp^*{}_2Ru_2)B_4H_{10}]$ (**I**), $[(Cp^*{}_2Cr_2)B_4H_8]$ (**II**) and $[(Cp^*{}_2Re_2)B_4H_8]$ (**III**).

Table S1. Experimental and calculated bond distances (Å) of **4-H**, **I**, **II** and **III**.

Bond distance	B1-B2		B2-B3		B3-B4		M1-M2	
Compounds	Exp.	Cal.	Exp.	Cal.	Exp.	Cal.	Exp.	Cal.
**4-H**	1.696(5)	1.700	1.799(6)	1.808	1.708(5)	1.700	3.6759(9)	3.647
**I**[18]	1.806(9)	1.800	1.784(10)	1.818	1.830(10)	1.801	2.8517(4)	2.864
**II**[19]	1.75(2)	1.803	1.75(2)	1.808	1.61(3)	1.803	2.870(2)	2.796
**III**[20]	1.74(5)	1.778	1.94(7)	2.744	1.64(5)	1.778	2.8092(7)	2.802

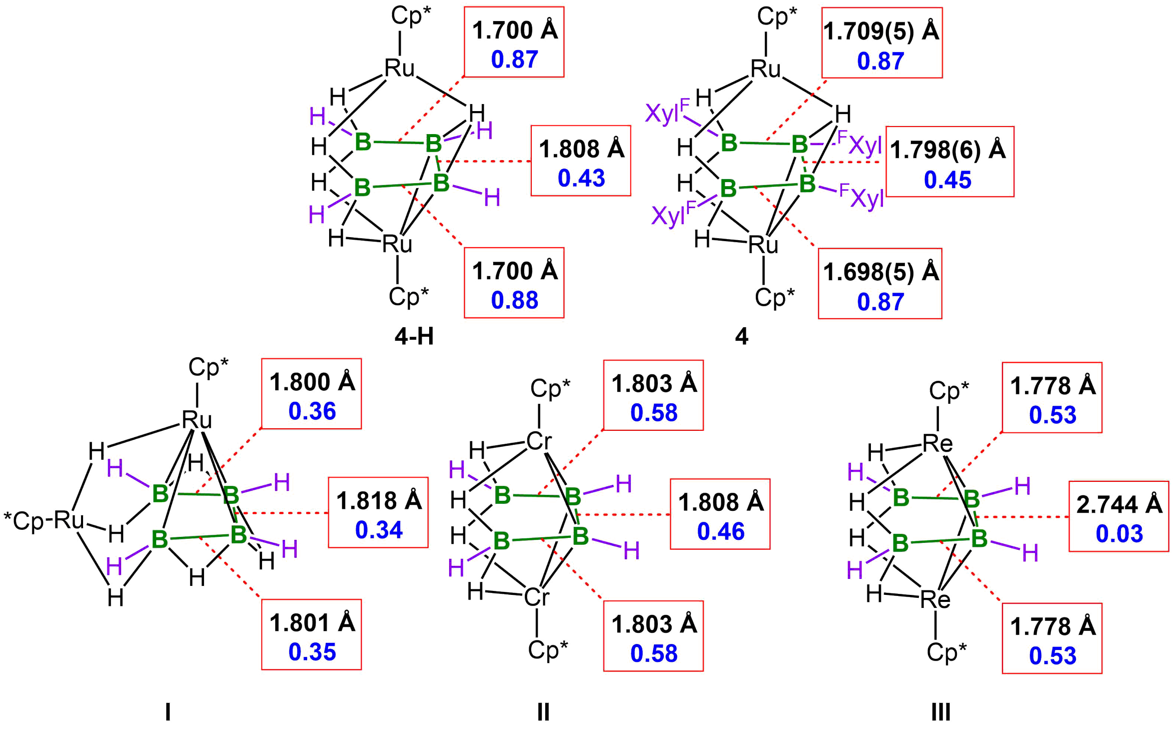

Figure S25. Mayer bond orders (blue) and distances for $[(Cp^*{}_2Ru_2)B_4H_9]^-$ (**4-H**), complex **4** (X-ray structure) and the literature-known complexes $[(Cp^*{}_2Ru_2)B_4H_{10}]$ (**I**), $[(Cp^*{}_2Cr_2)B_4H_8]$ (**II**) and $[(Cp^*{}_2Re_2)B_4H_8]$ (**III**).

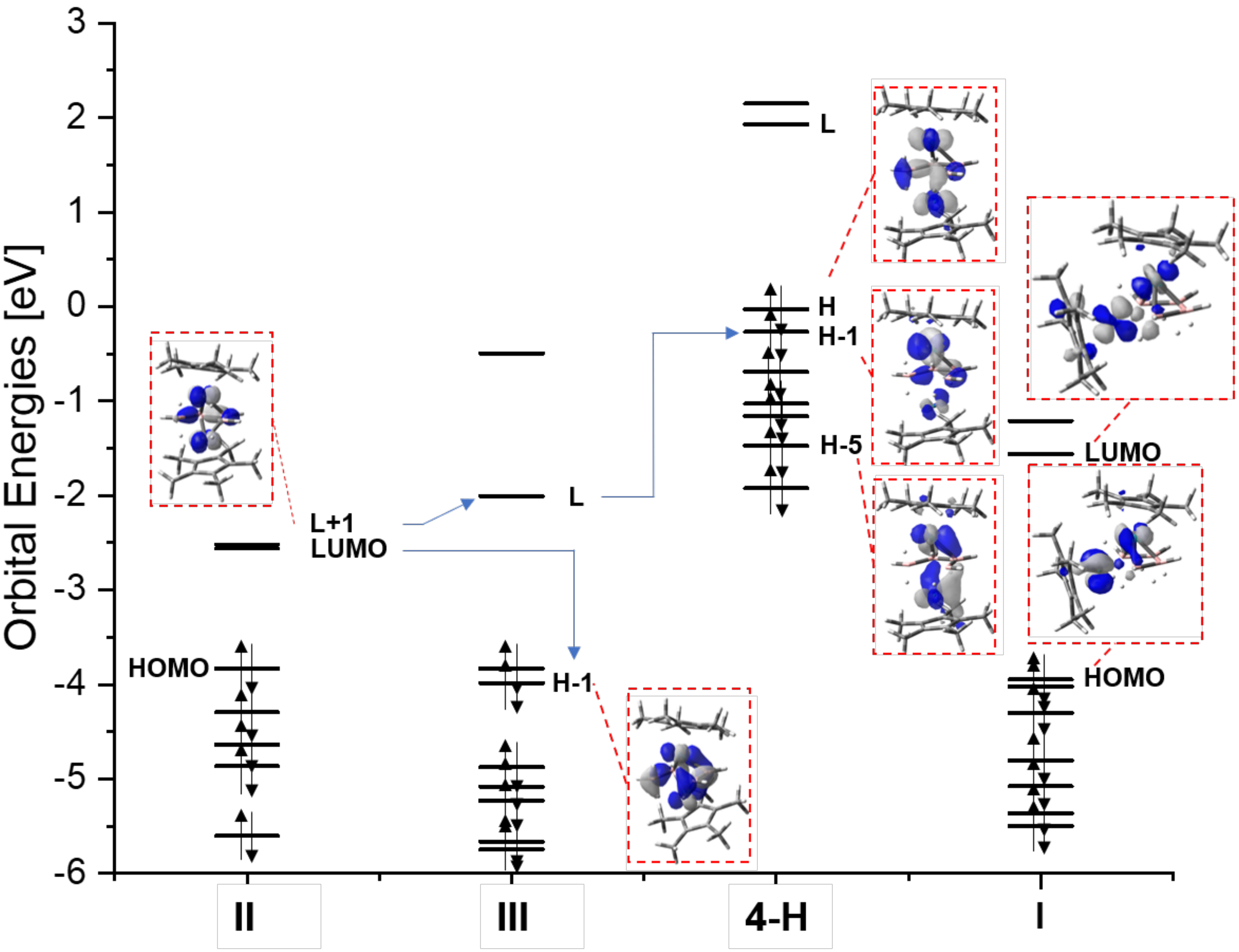

Figure S26. Frontier molecular orbitals (ZORA-BLYP-D3(BJ)/TZ2P) of metallaborane complexes **4-H**, **I**, **II** and **III**. Surface isovalue: ± 0.05 [$e\ a_0^{-3}$]^{½}. Note that HOMO, HOMO-1 and HOMO-5 are indentical in **4-H** and **4**.

XYZ Coordinates

4-H		NOIMAG	[-8139.01]
Ru	6.498373	10.992154	22.177609
C	4.279588	11.467355	22.138928
C	4.90556	11.954528	23.354624
C	5.906817	12.929744	22.973757
C	5.921733	13.014604	21.527669
C	4.541488	11.940334	19.573593
H	3.829018	12.712241	19.236781
H	4.08405	10.961888	19.392156
H	5.430643	12.009739	18.937747
C	3.147461	10.480813	22.085098
H	2.171777	10.976464	22.223525
H	3.249082	9.723378	22.869728
H	3.122124	9.956169	21.124039
C	4.509033	11.596701	24.758094
H	3.712828	12.257578	25.140951
H	5.367195	11.669763	25.433765
H	4.14578	10.564885	24.812284
C	6.719927	13.770702	23.914522
H	6.195287	14.706342	24.168232
H	7.687145	14.027125	23.471473
H	6.9261	13.228733	24.842645
C	6.764107	13.947296	20.705747
H	6.266301	14.919154	20.547852
H	6.981848	13.516235	19.722729
H	7.726096	14.132344	21.194235
C	4.90008	12.115338	21.022261
Ru	9.407969	8.822969	22.538196
C	10.808274	8.178077	24.079662
C	10.291711	7.017233	23.395706
C	10.645948	7.123171	21.990931
C	11.410012	8.347367	21.812842
C	11.501651	9.002621	23.095043
C	10.720702	8.447014	25.554424
H	11.568486	7.997857	26.099196
H	9.794548	8.039532	25.971906
H	10.719148	9.52199	25.760469
C	9.544502	5.882685	24.035493
H	10.233179	5.116625	24.428677
H	8.875905	5.396906	23.317324
H	8.924857	6.240084	24.864143
C	10.392226	6.083946	20.935837
H	11.212135	5.347007	20.889855
H	10.298906	6.542053	19.945845
H	9.463425	5.538777	21.133456
C	12.028783	8.824866	20.530738
H	12.063369	9.918313	20.496595
H	11.444472	8.494899	19.665779
C	12.248504	10.27265	23.386953
H	11.790953	10.818313	24.218497
H	12.242757	10.940451	22.520231
B	7.103453	8.961883	23.304423
H	6.216127	9.199585	22.372093
H	7.714288	8.093103	22.570155
B	7.893648	10.389276	23.780365
H	9.181576	10.562692	23.444894
B	8.686112	11.215574	22.38168
B	8.448687	10.367272	20.927585
H	7.174087	10.199411	20.67999
H	8.677739	9.097831	20.867686
H	13.297999	10.066654	23.655903
H	13.057295	8.445566	20.4107
H	7.94049	10.794685	24.916306
H	6.520411	8.352046	24.174027
H	8.921488	10.866617	19.930274
H	9.324146	12.235687	22.475936

I		NOIMAG	[-8211.45]
Ru	29.335499	22.772602	0.368026
Ru	26.777032	21.485714	0.342714
H	27.696151	22.835901	-0.257878
B	28.063476	22.349284	2.096516
B	29.781092	21.830332	2.236538
B	30.276298	20.893469	0.759117
B	28.901516	20.767132	-0.39715
H	27.207223	21.342994	2.018732
H	28.759894	21.540488	2.975136
H	30.090663	20.541645	2.038568
H	29.651087	19.850909	0.311728
H	27.841566	20.128583	0.050212
H	27.656143	23.183272	2.854859
H	30.613978	22.254263	2.977963
H	31.433937	20.706676	0.537437
H	29.137649	20.422475	-1.520404
C	29.229334	24.988959	-0.271584
C	30.178071	24.88297	0.789814
C	31.244361	24.017704	0.342596
C	30.966356	23.624165	-1.018937
C	29.719066	24.205456	-1.398607
C	28.011635	25.867361	-0.266624
H	27.265783	25.518672	-0.985267
H	28.272614	26.901196	-0.536059
H	27.540244	25.887545	0.720432
C	30.111706	25.585821	2.114456
H	30.62378	26.558265	2.069891
H	30.587134	24.993233	2.901484
H	29.075551	25.764815	2.416547
C	32.508965	23.722202	1.094501
H	32.34365	23.73597	2.17509
H	33.278776	24.471558	0.860715
H	32.907782	22.737992	0.832431
C	31.864556	22.808149	-1.901718
H	32.448201	22.089462	-1.31966
H	32.570389	23.454188	-2.444137
H	31.287246	22.245455	-2.641425
C	29.109325	24.136606	-2.769327
H	29.60287	24.842341	-3.453685
H	28.046833	24.38637	-2.747409
H	29.207176	23.133754	-3.197178
C	24.890907	22.644939	0.243693
C	24.657843	21.414178	0.960045
C	24.923559	20.307268	0.073715
C	25.310607	20.856536	-1.21569
C	25.278105	22.284106	-1.112243
C	24.620253	24.029854	0.754846
H	23.566577	24.312092	0.607344
H	25.238914	24.770479	0.240882
H	24.840269	24.105182	1.824139
C	24.161709	21.314147	2.373932
H	24.472835	20.375615	2.841843
H	23.063144	21.355029	2.398817
H	24.541916	22.136913	2.98646
C	24.7102	18.852192	0.379333
H	25.393782	18.219814	-0.195106
H	23.682928	18.546619	0.128986
H	24.872071	18.638372	1.44007
C	25.606336	20.063236	-2.454877
H	24.685201	19.857078	-3.019878
H	26.069411	19.10274	-2.209563
H	26.293593	20.600472	-3.115186
C	25.472955	23.241953	-2.249068
H	25.811486	24.218934	-1.89562
H	24.523256	23.394419	-2.782593
H	26.205053	22.867752	-2.969238

II		NOIMAG	[-8088.18]
Cr	17.247356	-0.508323	4.966276
Cr	19.079488	1.456339	4.190206
C	17.214914	-2.329013	6.247288
C	15.922526	-1.706932	6.296444
C	15.37324	-1.712892	4.974823
C	16.321491	-2.338574	4.098181
C	17.457069	-2.727333	4.888154
C	18.099995	-2.614669	7.425997
C	15.242474	-1.196055	7.536286
C	14.013541	-1.208643	4.577941
C	16.110807	-2.636801	2.641822
C	18.61379	-3.554589	4.410384
C	21.219386	1.957524	4.548815
C	21.121275	1.205374	3.328513
C	20.314077	1.953432	2.404715
C	19.926524	3.173971	3.05243
C	20.483369	3.17653	4.370886
C	22.039974	1.591863	5.7518
C	21.869873	-0.056312	3.015119
C	20.024232	1.582771	0.978894
C	19.126015	4.282124	2.426418
C	20.372527	4.287936	5.377431
B	17.224111	0.835095	3.275212
B	18.567918	-0.357529	3.428305
B	19.270444	-0.354123	5.094755
B	18.440068	0.840802	6.159517
H	17.83464	-3.576797	7.888554
H	18.007478	-1.838656	8.191745
H	19.150968	-2.660504	7.129046
H	14.666565	-1.99945	8.018251
H	15.967743	-0.824439	8.26606
H	14.5482	-0.38121	7.309465
H	13.268687	-2.014692	4.647645
H	13.68031	-0.392833	5.226613
H	14.008368	-0.840689	3.54776
H	15.598148	-3.601349	2.511832
H	15.500785	-1.866278	2.160919
H	17.062711	-2.684832	2.106657
H	18.378004	-4.624254	4.507224
H	19.516307	-3.35305	4.992869
H	18.84261	-3.352272	3.36096
H	21.594674	1.981661	6.672095
H	23.056758	2.003525	5.669642
H	22.121853	0.507186	5.859363
H	21.335312	-0.666085	2.282351
H	22.018164	-0.665348	3.910453
H	22.859097	0.185241	2.600088
H	20.006718	0.49765	0.848443
H	19.05304	1.969279	0.655596
H	20.790872	1.994215	0.305803
H	18.565459	4.848593	3.176273
H	19.788244	4.987183	1.9032
H	18.410268	3.894861	1.695374
H	19.442685	4.851775	5.255653
H	20.399714	3.904275	6.401523
H	21.207505	4.994575	5.264018
H	17.142769	0.628491	6.222907
H	20.17082	-1.063968	5.422926
H	18.962523	-1.069982	2.557106
H	18.474742	2.05729	5.658378
H	18.836497	0.91764	7.289443
H	17.605438	2.053142	3.596114
H	16.273306	0.624925	4.160593
H	16.691837	0.907608	2.202276

III		NOIMAG	[-8120.93]
RE	17.149254	-0.441362	4.899634
RE	19.0574	1.521252	4.303155
C	17.572807	-2.61374	5.572328
C	16.368685	-2.153784	6.217686
C	15.383064	-1.88664	5.198458
C	15.986666	-2.16688	3.920057
C	17.342458	-2.611574	4.148263
C	18.758762	-3.219058	6.26153
C	16.158576	-2.050013	7.702515
C	13.952869	-1.487698	5.432823
C	15.304512	-2.086669	2.583632
C	18.2726	-3.200099	3.128816
C	21.179609	2.197472	4.874108
C	21.292596	1.025446	4.036567
C	20.754149	1.355639	2.739665
C	20.294712	2.721378	2.784229
C	20.566183	3.247211	4.100061
C	21.685689	2.318546	6.283814
C	22.048696	-0.230305	4.356115
C	20.880005	0.513779	1.505363
C	19.708098	3.483739	1.629082
C	20.344938	4.664276	4.549929
B	16.933575	1.308672	3.384525
B	18.145169	0.039332	3.097032
B	19.090766	-0.092732	5.669301
B	18.019221	1.152111	6.350291
H	18.58655	-4.29337	6.424159
H	18.941288	-2.752728	7.232961

H	19.668445	-3.106274	5.666527
H	15.810531	-3.008147	8.11449
H	17.086652	-1.779163	8.213878
H	15.411547	-1.289413	7.948248
H	13.317689	-2.379188	5.542462
H	13.847858	-0.889737	6.34279
H	13.559596	-0.899612	4.598623
H	14.820862	-3.043014	2.337662
H	14.535107	-1.309083	2.573539
H	16.020026	-1.851817	1.791414
H	18.167403	-4.29483	3.106998
H	19.315226	-2.965826	3.363667
H	18.064224	-2.817278	2.127878
H	21.095282	3.036858	6.860211
H	22.731546	2.657776	6.292263
H	21.633723	1.358143	6.80313
H	21.584238	-1.102002	3.885544
H	22.084379	-0.414044	5.431667
H	23.081759	-0.154901	3.985864
H	20.837335	-0.552377	1.741704
H	20.080293	0.724517	0.790893
H	21.84224	0.717316	1.012439
H	19.0363	4.275907	1.972764
H	20.501178	3.952709	1.029111
H	19.132859	2.824155	0.973199
H	19.489507	5.117307	4.040419
H	20.160226	4.718176	5.626671
H	21.229902	5.280075	4.33112
H	16.769953	0.88758	6.212143
H	19.931161	-0.717675	6.229097
H	18.383783	-0.524588	2.079503
H	18.141335	2.297279	5.780858
H	18.267474	1.268906	7.516228
H	17.430802	2.40109	3.841591
H	16.061249	0.989307	4.272186
H	16.37191	1.536805	2.351499

Crystallographic Details

The crystal data of **1a**, **3a**, were collected on a Bruker X8-APEX II diffractometer with a CCD area detector, the crystal data of **1b**, **2a**, **4** and **5** were collected on a BRUKER D8 QUEST diffractometer with a CMOS area detector and multi-layer mirror monochromated $Mo_{K\alpha}$ radiation. The structures were solved using the intrinsic phasing method,[21] refined with the ShelXL program[22] and expanded using Fourier techniques. All non-hydrogen atoms were refined anisotropically. Hydrogen atoms were included in the structure factor calculations. All hydrogen atoms were assigned to idealized geometric positions.

[(Cp*RuH)$_2$BDur] (1a). All hydrogen atoms located next to ruthenium metal or located on boron were assigned to idealized positions. The crystal was a pseudo-merohedral twin with domains rotated by 180° around real axis [1.000 0.00 0.00]. The BASF parameter was refined to 40%. The distances between atoms the bridging hydrogen atoms and the ruthenium atoms were kept at the value of 1.8 (Ru1/2_1/11-H1/2_1/11) 2.276 (H1_1/11-H2_1/11) during refinement using DFIX restraint. The distances between atoms the boron atom and the bridging hydrides were restrained during refinement to the same value with SADI restraint. Crystal data: $C_{30}H_{45}BRu_2$, M_r = 618.61, yellow plate, 0.17×0.11×0.1 mm^3, triclinic space group $P\bar{1}$, a = 9.0544(4) Å, b = 16.0188(7) Å, c = 19.7565(9) Å, α = 79.476(2)°, β = 89.887(2)°, γ = 79.142(2)°, V = 2765.3(2) Å^3, Z = 4, ρ_{calcd} = 1.486 g·cm^{-3}, μ = 1.108 mm^{-1}, F(000) = 1272, T = 100(2) K, R_1 = 0.0476, wR^2 = 0.0970, 14311 independent reflections [$2\theta \leq 59.756°$] and 636 parameters. CCDC-1901322.

[(Cp*RuH)$_2$BN(TMS)$_2$] (1b). All hydrogen atoms except the bridging hydrogen atoms H3_1 and H4_1 were assigned to idealized positions. The coordinates of H3_1 and H4_1 were refined freely. The U_{ii} displacement parameters of atoms C1_5/6 to C10_5/6 (residues 5 and 6 Cp*) were restrained with the ISOR keyword to approximate isotropic behavior. Crystal data: $C_{26}H_{50}BNRu_2Si_2$, M_r = 645.80, yellow plate, 0.015×0.015×0.01 mm^3, orthorhombic space group $P2_12_12_1$, a = 8.721(6) Å, b = 10.266(8) Å, c = 33.47(3) Å, V = 2996(4) Å^3, Z = 4, ρ_{calcd} = 1.432 g·cm^{-3}, μ = 1.102 mm^{-1}, F(000) = 1336, T = 100(2) K, R_1 = 0.0631, wR^2 = 0.0637, 6339 independent reflections [$2\theta \leq 53.514°$] and 313 parameters. CCDC-1901321.

[(Cp*Ru)$_2$(μ-H)(μ-κ^3-H,H,H-H$_3$BDur)] (2a). All hydrogen atoms except the bridging hydrogen atoms (H1_1, H2_1, H3_1 and H4_1) were assigned to idealized positions. The coordinates of those hydrogen atoms were refined freely. The displacement parameters of atoms in disordered Cp*-Ligand (C1_3 to C19_3) were restrained to the same value with similarity restraint SIMU. In addition the U_{ii} displacement parameters of those atoms were restrained with ISOR keyword to

approximate isotropic behavior, and with RIGU keyword in ShelXL input ('enhanced rigid bond' restraint for all bonds in the connectivity list. Standard values of 0.01 for both parameters s1 and s2 were used). Crystal data: $C_{30}H_{47}BRu_2$, M_r = 620.62, yellow plate, 0.12×0.09×0.08 mm^3, monoclinic space group $P2_1/c$, a = 9.058(6) Å, b = 21.491(15) Å, c = 14.900(10) Å, β = 91.81(3)°, V = 2899(3) Å^3, Z = 4, ρ_{calcd} = 1.422 g·cm^{-3}, μ = 1.057 mm^{-1}, F(000) = 1280, T = 100(2) K, R_1 = 0.0470, wR^2 = 0.0857, 5708 independent reflections [2θ ≤ 52.04°] and 419 parameters. CCDC-1901326.

[Cp*Ru{κ^3-H,H,H-(H_3BDur)}] (3a). The U_{ii} displacement parameters of atoms C2 were restrained with ISOR 0.05 keyword to approximate isotropic behavior due to minor rotational disorder. Crystal data: $C_{20}H_{31}BRu$, M_r = 383.33, orange block, 0.7×0.6×0.14 mm^3, monoclinic space group $P2_1/n$, a = 14.0513(11) Å, b = 9.0914(6) Å, c = 15.5354(11) Å, β = 105.009(2)°, V = 1916.9(2) Å^3, Z = 4, ρ_{calcd} = 1.328 g·cm^{-3}, μ = 0.813 mm^{-1}, F(000) = 800, T = 100(2) K, R_1 = 0.0562, wR^2 = 0.1057, 4087 independent reflections [2θ ≤ 53.496°] and 220 parameters. CCDC-1901323.

Li(THF)$_4$[(Cp*Ru)$_2$B$_4$Ar$_4$H$_5$] (4). All hydrogen atoms except the those bound to boron or ruthenium were assigned to idealized positions and their coordinates were refined freely. The displacement parameters of atoms in disordered CF_3 groups were constrained to the same value with the EADP keyword. The central carbon atoms in both parts of the disordered CF_3 group were constrained to the same position. The displacement parameters of atoms in the disordered CF_3 as well as the disordered THF molecule were restrained to the same value with similarity restraint SIMU and the U_{ii} displacement parameters were restrained with the ISOR keyword to approximate isotropic behavior. Additionally, the atomic displacement parameters of atoms were restrained with the RIGU keyword in the ShelXL input ('enhanced rigid bond' restraint for all bonds in the connectivity list. Standard values of 0.004 for both parameters s1 and s2 were used). The 1-2 and 1-3 distances in the disordered THF molecule were restrained to the same values with SAME. Crystal data: $C_{68}H_{79}B_4F_{24}LiO_4Ru_2$, M_r = 1668.63, red block, 0.383×0.184×0.14 mm^3, orthorhombic space group $P2_12_12_1$, a = 12.595(4) Å, b = 18.767(5) Å, c = 30.916(6) Å, V = 7308(3) Å^3, Z = 4, ρ_{calcd} = 1.517 g·cm^{-3}, μ = 0.520 mm^{-1}, F(000) = 3384, T = 100(2) K, R_1 = 0.0269, wR^2 = 0.0660, 14385 independent reflections [2θ ≤ 52.044°] and 964 parameters. CCDC-1901327.

[(Cp*Ru)$_2${μ-η^2:η^2-B$_2$H$_2$(3,5-(CF$_3$)$_2$C$_6$H$_3$)$_2$}$_2$] (5). All hydrogen atoms except those bound to boron atoms were assigned to idealized positions. The coordinates of those hydrogen atoms were refined freely. The displacement parameters of atoms in the disordered CF_3 residues were constrained to the same value with EADP keyword. The displacement parameters of those atoms were restrained to the same value with similarity restraint SIMU and the U_{ii} displacement parameters were

restrained with the ISOR keyword to approximate isotropic behavior. A few of those atoms required a restraint with the RIGU keyword in ShelXL input ('enhanced rigid bond' restraint for all bonds in the connectivity list. Standard values of 0.004 for both parameters s1 and s2 were used). Crystal data: $C_{52}H_{46}B_4F_{24}Ru_2$, M_r = 1372.27, orange block, 0.326×0.15×0.111 mm^3, triclinic space group $P\,\bar{1}$, a = 12.806(4) Å, b = 12.874(4) Å, c = 18.308(6) Å, α = 74.912(12)°, β = 72.123(10)°, γ = 79.048(12)°, V = 2753.5(15) Å^3, Z = 2, ρ_{calcd} = 1.655 g·cm^{-3}, μ = 0.666 mm^{-1}, F(000) = 1364, T = 100(2) K, R_1 = 0.0556, wR^2 = 0.0941, 10831 independent reflections [$2\theta \leq$ 52.042°] and 908 parameters. CCDC-1901324.

References

[1] C. M. Fendrick, L. D. Schertz, E. A. Mintz, T. J. Marks, *Inorg. Synth.* **1992**, *29*, 193–198.

[2] U. Koelle, J. Kossakowski, *Inorg. Synth.* **1992**, *29,* 225–228.

[3] H. Suzuki, H. Omori, D.-H. Lee, Y. Yoshida, M. Fukushima, M. Tanaka, Y. Moro-oka, *Organometallics* **1994**, *13*, 1129–1146.

[4] N. Arnold, S. Mozo, U. Paul, U. Radius, H. Braunschweig, *Organometallics* **2015**, *34*, 5709−5715.

[5] W. R. Nutt, R. L. Wells, *Inorg. Chem.* **1982**, *21*, 2469–2473.

[6] K. Samigullin, M. Bolte, H.-W. Lerner, M. Wagner, *Organometallics* **2014**, *33*, 3564−3569.

[7] a) E. J. Baerends, T. Ziegler, A. J. Atkins, J. Autschbach, O. Baseggio, D. Bashford, A. Bérces, F. M. Bickelhaupt, C. Bo, P. M. Boerrigter, L. Cavallo, C. Daul, D. P. Chong, D. V. Chulhai, L. Deng, R. M. Dickson, J. M. Dieterich, D. E. Ellis, M. van Faassen, L. Fan, T. H. Fischer, C. Fonseca Guerra, M. Franchini, A. Ghysels, A. Giammona, S. J. A. van Gisbergen, A. Goez, A. W. Götz, J. A. Groeneveld, O. V. Gritsenko, M. Grüning, S. Gusarov, F. E. Harris, P. van den Hoek, Z. Hu, C. R. Jacob, H. Jacobsen, L. Jensen, L. Joubert, J. W. Kaminski, G. van Kessel, C. König, F. Kootstra, A. Kovalenko, M. V. Krykunov, E. van Lenthe, D. A. McCormack, A. Michalak, M. Mitoraj, S. M. Morton, J. Neugebauer, V. P. Nicu, L. Noodleman, V. P. Osinga, S. Patchkovskii, M. Pavanello, C. A. Peeples, P. H. T. Philipsen, D. Post, C. C. Pye, H. Ramanantoanina, P. Ramos, W. Ravenek, J. I. Rodríguez, P. Ros, R. Rüger, P. R. T. Schipper, D. Schlüns, H. van Schoot, G. Schreckenbach, J. S. Seldenthuis, M. Seth, J. G. Snijders, M. Solà, M. Stener, M. Swart, D. Swerhone, V. Tognetti, G. te Velde, P. Vernooijs, L. Versluis, L. Visscher, O. Visser, F. Wang, T. A. Wesolowski, E. M. van Wezenbeek, G. Wiesenekker, S. K. Wolff, T. K. Woo, A. L. Yakovlev, ADF2016, SCM, Theoretical Chemistry, Vrije Universiteit, http://www.scm.com, Amsterdam, The Netherlands, **2016**; b) C. Fonseca Guerra, J. G. Snijders, G. te Velde, E. J. Baerends, *Theor. Chem. Acc.* **1998**, *99*, 391-403; c) G. te Velde, F. M. Bickelhaupt, E. J. Baerends, C. Fonseca Guerra, S. J. A. van Gisbergen, J. G. Snijders, T. Ziegler, *J. Comput. Chem.* **2001**, *22*, 931-967.

[8] a) A. D. Becke, *J. Chem. Phys.* **1988**, *88*, 2547-2553; b) M. Franchini, P. H. T. Philipsen, L. Visscher, *J. Comput. Chem.* **2013**, *34*, 1819-1827.

[9] E. Van Lenthe, E. J. Baerends, *J. Comput. Chem.* **2003**, *24*, 1142-1156.

[10] J. C. Slater, *Quantum Theory of Molecules and Solids*, 4 ed., McGraw-Hill, New York, **1974**.

[11] a) A. D. Becke, *J. Chem. Phys.* **1986**, *84*, 4524; b) A. D. Becke, *Phys. Rev. A* **1988**, *38*, 3098-3100.

[12] a) T. V. Russo, R. L. Martin, P. J. Hay, *J. Chem. Phys.* **1994**, *101*, 7729-7737; b) B. G. Johnson, P. M. W. Gill, J. A. Pople, *J. Chem. Phys.* **1993**, *98*, 5612-5626; c) C. Lee, W. Yang, R. G. Parr, *Phys. Rev. B* **1988**, *37*, 785-789.

[13] a) E. v. Lenthe, E. J. Baerends, J. G. Snijders, *J. Chem. Phys.* **1993**, *99*, 4597-4610; b) E. van Lenthe, E. J. Baerends, J. G. Snijders, *J. Chem. Phys.* **1994**, *101*, 9783-9792; c) E. van Lenthe, J. G. Snijders, E. J. Baerends, *J. Chem. Phys.* **1996**, *105*, 6505-6516.

[14] S. Grimme, S. Ehrlich, L. Goerigk, *J. Comput. Chem.* **2011**, *32*, 1456-1465.

[15] a) A. Berces, R. M. Dickson, L. Y. Fan, H. Jacobsen, D. Swerhone, T. Ziegler, *Comput. Phys. Commun.* **1997**, *100*, 247-262; b) H. Jacobsen, A. Berces, D. P. Swerhone, T. Ziegler, *Comput. Phys. Commun.* **1997**, *100*, 263-276; c) S. K. Wolff, *Int. J. Quantum Chem.* **2005**, *104*, 645-659.

[16] I. Mayer, *J. Comput. Chem.* **2007**, *28*, 204-221.

[17] F. M. Bickelhaupt, N. J. R. van Eikema Hommes, C. Fonseca Guerra, E. J. Baerends, *Organometallics* **1996**, *15*, 2923-2931.

[18] M. A. Peldo, A. M. Beatty, T. P. Fehlner, *Organometallics* **2003**, *22*, 3698-3702.

[19] S. Ghosh, M. Shang, T. P. Fehlner, *J. Organomet. Chem.* **2000**, *614-615*, 92-98.

[20] K. J. Deck, Y. Nishihara, M. Shang, T. P. Fehlner, *J. Am. Chem. Soc.* **1994**, *116*, 8408–8409.

[21] G. Sheldrick, *Acta Cryst.* **2015**, *A71*, 3–8.

[22] G. Sheldrick, *Acta Cryst.* **2008**, *A64*, 112–122.
